# Piccolo Promotes Vesicle Replenishment at a Fast Central Auditory Synapse

**DOI:** 10.3389/fnsyn.2017.00014

**Published:** 2017-10-25

**Authors:** Tanvi Butola, Carolin Wichmann, Tobias Moser

**Affiliations:** ^1^Institute for Auditory Neuroscience and InnerEarLab, University Medical Center Göttingen, Göttingen, Germany; ^2^Göttingen Graduate School for Neurosciences, Biophysics, and Molecular Biosciences (GGNB), University of Göttingen, Göttingen, Germany; ^3^International Max Planck Research School for Neurosciences (IMPRS), Göttingen, Germany; ^4^Synaptic Nanophysiology Group, Max Planck Institute for Biophysical Chemistry (MPG), Göttingen, Germany; ^5^Collaborative Research Centers 889 and 1286, University of Göttingen, Göttingen, Germany; ^6^Molecular Architecture of Synapses Group, Center for Biostructural Imaging of Neurodegeneration (BIN), University of Göttingen, Göttingen, Germany; ^7^Center for Nanoscale Microscopy and Molecular Physiology of the Brain, University of Göttingen, Göttingen, Germany

**Keywords:** endbulb of Held, cochlear nucleus, readily releasable pool, short-term depression, Bassoon

## Abstract

Piccolo and Bassoon are the two largest cytomatrix of the active zone (CAZ) proteins involved in scaffolding and regulating neurotransmitter release at presynaptic active zones (AZs), but have long been discussed as being functionally redundant. We employed genetic manipulation to bring forth and segregate the role of Piccolo from that of Bassoon at central auditory synapses of the cochlear nucleus—the endbulbs of Held. These synapses specialize in high frequency synaptic transmission, ideally poised to reveal even subtle deficits in the regulation of neurotransmitter release upon molecular perturbation. Combining semi-quantitative immunohistochemistry, electron microscopy, and *in vitro* and *in vivo* electrophysiology we first studied signal transmission in Piccolo-deficient mice. Our analysis was not confounded by a cochlear deficit, as a short isoform of Piccolo (“Piccolino”) present at the upstream ribbon synapses of cochlear inner hair cells (IHC), is unaffected by the mutation. Disruption of Piccolo increased the abundance of Bassoon at the AZs of endbulbs, while that of RIM1 was reduced and other CAZ proteins remained unaltered. Presynaptic fiber stimulation revealed smaller amplitude of the evoked excitatory postsynaptic currents (eEPSC), while eEPSC kinetics as well as miniature EPSCs (mEPSCs) remained unchanged. Cumulative analysis of eEPSC trains indicated that the reduced eEPSC amplitude of Piccolo-deficient endbulb synapses is primarily due to a reduced readily releasable pool (RRP) of synaptic vesicles (SV), as was corroborated by a reduction of vesicles at the AZ found on an ultrastructural level. Release probability seemed largely unaltered. Recovery from short-term depression was slowed. We then performed a physiological analysis of endbulb synapses from mice which, in addition to Piccolo deficiency, lacked one functional allele of the Bassoon gene. Analysis of the double-mutant endbulbs revealed an increase in release probability, while the synapses still exhibited the reduced RRP, and the impairment in SV replenishment was exacerbated. We propose additive roles of Piccolo and Bassoon in SV replenishment which in turn influences the organization and size of the RRP, and an additional role of Bassoon in regulation of release probability.

## Introduction

**Figure 1:**
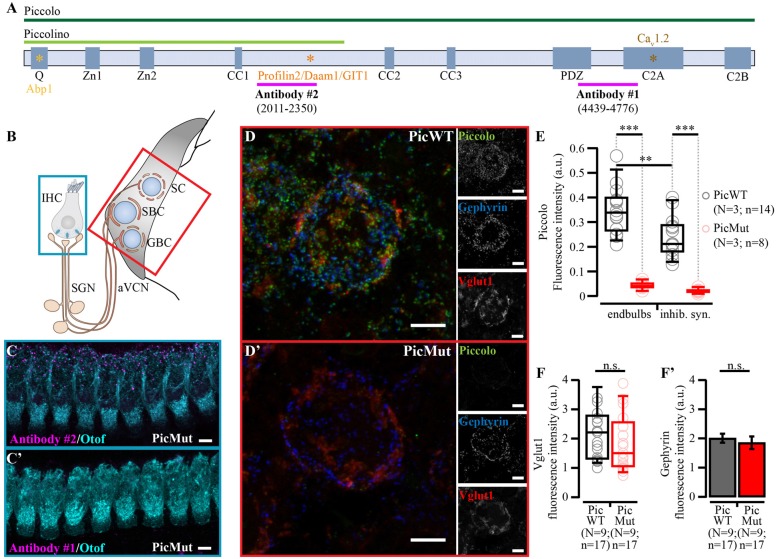
Selective expression of Piccolo (Aczonin) in central synapses. **(A)** Domain structure of Piccolo (dark green line) and its shorter isoform Piccolino (light green line). Magenta lines illustrate the position of antigenic peptides used to raise Piccolo antibodies employed in this study. Antibody #1 binding to the C-terminus, selectively identifies Piccolo, while antibody #2 binding to the central region recognizes both Piccolo and Piccolino. Colored asterisks denote binding domains unique to Piccolo (not present in Bassoon) with their respective binding partners written in the same color (see “Introduction” section for description). **(B)** Scheme of the site of investigation (not drawn to scale): the endbulbs of Held are formed by the auditory nerve fibers (central neurites of SGN) on bushy cells (BCs) of the anterior ventral cochlear nucleus (aVCN; red box). SGNs receive their input from ribbon-type active zones (AZs) of inner hair cells (IHCs, blue box) of the organ of Corti. Spherical bushy cells (SBC) and gobular bushy cells (GBC) receive different numbers of endbulbs. SGNs form bouton-like synapses on stellate cells (SC). **(C)** Preservation of Piccolino in the organ of Corti of PicMut mice: Maximal projection of confocal images show immunofluorescent puncta of Piccolino (**C**, antibody #2) in otoferlin (Otof)-labeled IHCs of the organ of Corti, while no Piccolo signal (**C’**, antibody #1) is found. **(D)** Reduced expression of Piccolo in PicMut: Maximum projection of confocal image stack of a bushy cell in PicWT **(D)** and PicMut **(D’)** labeled for Piccolo (antibody #1), Vglut1 (excitatory synapses) and Gephyrin (inhibitory synapses). **(E)** Reduced fluorescence intensity of Piccolo (antibody #1) puncta at the BCs of aVCN, at both the endbulb AZs and the inhibitory AZs (inhib. syn.), in PicMut (*N* = 3; *n* = 8) mice as compared to PicWT (*N* = 3; *n* = 14) mice as obtained in maximum projections of confocal images. ***p-value < 0.001, **p-value < 0.01, Wilcoxon rank sum test. **(F)** Unaltered fluorescence intensity of Vglut1 (**F**, staining excitatory synapses) and Gephyrin (**F’**, staining inhibitory synapses) in the confocal images used to analyze molecular composition of cytomatrix of the AZ (CAZ) proteins at BCs in the aVCN (n.s. *p*-value ≥ 0.05, Wilcoxon signed rank test in **F**, paired Student’s *t*-test in **F’**). Data information: Box and whisker plots present median, lower/upper quartiles and 10–90th percentiles. Bar plot represents mean ± SEM (error bars). *N*, number of animals; *n*, number of BCs. All scale bars −5 μm.

Active zones (AZs) are specialized regions at the presynaptic terminals where neurotransmitter release occurs. AZs feature an electron-dense meshwork of proteins called the cytomatrix of the active zone (CAZ). CAZ comprises of multi-domain protein families like: Munc13s, Rab3-interacting molecules (RIMs), RIM-binding proteins (RIM-BPs), CAST/ELKS proteins, Piccolo/Aczonin and Bassoon, and Liprins-α (Schoch and Gundelfinger, 2006; Gundelfinger and Fejtova, 2012; Südhof, 2012). The two largest members (>400 kDa) of the CAZ, Piccolo (Fenster et al., 2000) and Bassoon (tom Dieck et al., 1998), are vertebrate-specific and structurally similar. They play an integral role in AZ assembly and scaffolding (Südhof, 2012; Gundelfinger et al., 2016), synaptic vesicle (SV) clustering (Mukherjee et al., 2010), presynaptic protein ubiquitination and degradation (Waites et al., 2013), and CtBP1-mediated activity-regulated gene expression via synapse-to-nucleus signaling (Ivanova et al., 2015, 2016). Piccolo (Figure 1A) and Bassoon, share 10 highly conserved regions, Piccolo Bassoon Homology domains (PBH; tom Dieck et al., 1998; Wang et al., 1999; Fenster et al., 2000; Schoch and Gundelfinger, 2006) including Zn finger and coiled-coiled (CC) domains, which might explain partial overlap in their function.

However, despite their close homology, Piccolo has additional features that may ascribe unique functions to it, divergent from Bassoon (Gundelfinger et al., 2016). Piccolo has a unique segment between its first and second CC domains (Figure 1A, orange*), selectively interacting with Actin-associated proteins like Daam1 (Wagh et al., 2015) and Profilin2 (Wang et al., 1999; Waites et al., 2011), thought to regulate dynamic assembly of F-actin within the presynaptic terminal (Waites et al., 2011; Wagh et al., 2015). Since Actin has been a long standing candidate for regulation of SV dynamics involved in exocytosis and endocytosis (Sakaba and Neher, 2003; Lee et al., 2012; Nguyen et al., 2012; Watanabe et al., 2013; Delvendahl et al., 2016; Soykan et al., 2017), Piccolo’s interaction with it might indicate a role of Piccolo in translocating SVs within the nerve terminal thereby regulating SV dynamics and synaptic transmission. The same region between the two CC domains of Piccolo (Figure 1A, orange*), also binds GIT protein (Kim et al., 2003), which interacts with endocytic adaptor protein Stonin2 (Podufall et al., 2014). Another interaction unique to Piccolo is of its N-terminal glutamine-rich (Poly Q; Figure 1A, yellow*) motif with Abp1 (Fenster et al., 2003), which in turn associates with endocytic GTPase Dynamin (Kessels et al., 2001). These interactions with Abp1 and GIT provide additional links to Piccolo’s role in vesicle endocytosis. Unlike Bassoon, Piccolo additionally has a C-terminal PDZ domain and two C-terminal C_2_ domains (C_2_A and C_2_B). The PDZ domain has been linked to exocytosis in pancreatic β-cells (Fujimoto et al., 2002; Shibasaki et al., 2004). The C_2_A domain (Figure 1A, brown*) was reported to bind to Ca_v_1.2 L-type voltage-dependent Ca^2+^ channels (Shibasaki et al., 2004), and may act as a low-affinity Ca^2+^ sensor for exocytosis, making Piccolo a candidate for detecting Ca^2+^ build-up during high frequency stimulation (Gerber et al., 2001; Garcia et al., 2004; Schoch and Gundelfinger, 2006). While several hypotheses have been put forward for the function of Piccolo, it has remained challenging to unravel its physiological role(s). One study based on RNAi implicated Piccolo as an inhibitor of exocytosis (Leal-Ortiz et al., 2008), while the other, that generated the mouse mutant (Mukherjee et al., 2010) employed in our study, revealed a role in SV clustering in conjunction with Bassoon, but failed to unmask any major exocytosis phenotype.

Here, we studied the consequences of genetic Piccolo disruption at the first central auditory synapse—the endbulb of Held synapse (von Gersdorff and Borst, 2002; Yu and Goodrich, 2014), formed by the spiral ganglion neurons (SGNs) synapsing onto the bushy cells (BCs) of the anterior ventral cochlear nucleus (aVCN; Figure 1B). These large calyceal synapses typically employ more than 100 AZs for reliable and temporally precise signal transmission at frequencies of hundreds of Hertz (Trussell, 1999; Wang et al., 2011). Given the high functional demand, these synapses seem ideally poised for unveiling any discrepancies in synaptic transmission due to molecular perturbation. We combined electrophysiological analysis with studies of the molecular composition and ultrastructure of the AZ in endbulbs of Piccolo-deficient mice. Our results indicate a role of Piccolo in promoting SV replenishment to the readily releasable pool (RRP) and a, likely compensatory, up-regulation of Bassoon at Piccolo-deficient synapses.

Apart from focusing on bringing forth the unique function(s) of Piccolo at the AZ, we investigated the changes in synaptic transmission, with an additional Bassoon manipulation (Altrock et al., 2003). Unlike Piccolo, Bassoon has been the focus of extensive investigation concentrating solely on its function and not just its role in conjunction with Piccolo (Mukherjee et al., 2010; Waites et al., 2013; Ivanova et al., 2016).

In sensory synapses, Bassoon is essential for tethering the synaptic ribbon at AZs both at retinal photoreceptors (Dick et al., 2003; tom Dieck et al., 2005) and cochlear inner hair cells (IHC; Khimich et al., 2005). Bassoon was also found to maintain the Ca^2+^ channel clustering and synaptic vesicle pool at the presynapse (Frank et al., 2010), and hence establish reliable signal transmission to the auditory nerve fibers (Buran et al., 2010; Jing et al., 2013). In central synapses, Bassoon has been shown to localize P/Q type Ca^2+^ channels at the AZ (Davydova et al., 2014), and its absence was associated with increase in the number of silent synapses in hippocampal cultures (Altrock et al., 2003), and impaired vesicle replenishment (Hallermann et al., 2010; Mendoza Schulz et al., 2014). Most recently, Bassoon was proposed to be a regulator of presynaptic autophagy (Okerlund et al., 2017).

In context of the extensive research on the function of Bassoon and its role together with Piccolo, we first focused on the function of Piccolo in synaptic transmission. Since the two proteins might compensate for each other’s absence (Mendoza Schulz et al., 2014), studying Piccolo deficiency at the synapse, followed by a partial Bassoon loss in addition aimed to resolve the non-overlapping functions of the proteins. Our work reveals that Piccolo and Bassoon synergistically promote vesicle replenishment and determine the RRP of synaptic vesicles (SV), but possibly employ unique means to this common goal. Piccolo seems not to influence release probability, while Bassoon may be a regulator of it.

## Materials and Methods

### Animals

Mice with cre-mediated excision (Lakso et al., 1996) of exon 14 of the *Pclo* gene and insertion of a neomycin resistance cassette in the adjacent 3′ intron (Mukherjee et al., 2010; PicMut), and their wildtype littermates (PicWT), of either sex, were studied from postnatal day 14–23. The mouse line was derived by heterozygous breeding with C57Bl/6J genetic background. Animals were genotyped, and re-genotyped post experiments, using PCR. PicBsn animals, with only one intact allele of the *Bsn* gene in addition to Piccolo mutation, were used. These were derived by heterozygous breeding of PicMut with *Bsn*^ΔEx4/5^ mice (exons 4 and 5 of Bassoon gene deleted (Altrock et al., 2003). All experiments were performed in compliance with the guidelines of the German animal welfare act and were approved by the board for animal welfare of the University Medical Center Göttingen and the animal welfare office of the state of Lower Saxony.

### *In Vitro* Electrophysiology

#### Slice Preparation

Acute parasagittal slices (150 μm) from the cochlear nucleus were obtained as described previously (Mendoza Schulz et al., 2014). Briefly, after sacrifice by decapitation, brains were dissected out and quickly immersed in ice-cold low Na^+^ and low Ca^2+^ cutting solution containing (in mM): 50 NaCl, 26 NaHCO_3_, 120 sucrose, 1.25 NaH_2_PO_4_.H_2_O, 2.5 KCl, 20 glucose, 0.2 CaCl_2_, 6 MgCl_2_, 0.7 Na L-ascorbate, 2 Na pyruvate, 3 myo-inositol, 3 Na L-lactate with pH adjusted to 7.4 and osmolarity of around 310 mOsm/l. After removal of the meninges from the ventral face of the brainstem, the two hemispheres were separated by a midsagittal cut and the forebrain was removed at the pons-midbrain junction. The brain blocks containing brain stem and cerebellum were then glued (cyanoacrylate glue; Loctite 401, Henkel) to the stage of a VT 1200S vibratome (Leica microsystems, Wetzlar, Germany) such that the medial side was glued on, the ventral side was facing the blade and the lateral side was facing upwards, submerged in ice-cold cutting solution. For sectioning, the blade was positioned at the height of cerebellar flocculus and sections were cut at a blade feed rate of 0.02 mm/s with an amplitude of 1.50 mm. Slices were incubated for 30 min in artificial cerebrospinal fluid (aCSF) maintained at 35°C, and then kept at room temperature (22–24°C) until recording. Composition of aCSF was identical to the cutting solution except (in mM): 125 NaCl, 13 glucose, 1.5 CaCl_2_ and 1 MgCl_2_. The pH of the solution was adjusted to 7.4 and osmolarity was around 310 mOsm/l. All solutions were continuously aerated with carbogen (95% O_2_, 5% CO_2_).

#### Electrophysiology

Patch-clamp recordings were made from BCs of aVCN using EPC10 USB Patch clamp amplifier controlled by the Patchmaster software (HEKA Elektronik, Lambrecht/Pfalz, Germany). Sampling interval and filter settings were 25 μs and 7.3 kHz respectively. Cells were visualized by differential interference contrast (DIC) microscopy through a 40× water-immersion objective (NA 0.8; Zeiss, Oberkochen, Germany) using an Axioscope2 FS plus microscope (Zeiss, Oberkochen, Germany). All experiments were conducted at a temperature of 33–35°C, maintained by constant superfusion (flow rate 3–4 ml/min) of aCSF, heated by an inline solution heater (SH-27B with TC-324B controller; Warner Instruments, Hamden, CT, USA) and monitored by a thermistor placed between the inflow site and the slice in the recording chamber.

Patch pipettes were pulled with P-87 micropipette puller (Sutter Instruments Co., Novato, CA, USA) from borosilicate glass capillaries with filament (GB150F, 0.86 × 1.50 × 80 mm; Science Products, Hofheim, Germany). Open tip pipette resistance was 1.5–3 MΩ when filled with intracellular solution containing (in mM): 115 K-gluconate, 10 HEPES, 8 EGTA, 10 Na_2_Phosphocreatine, 4 ATP-Mg, 0.3 GTP-Na, 4.5 MgCl_2_, 10 NaCl and 1 *N*-(2, 6-dimethylphenyl carbamoylmethyl)triethylammonium chloride (QX-314; Alomone Labs, Jerusalem, Israel) to block sodium channels, with a pH of 7.35 and an osmolarity of 300 mOsm/l. Additionally, 1 mM of fluorescent dye Alexa-488 (Invitrogen) was added to the recording pipette and cell structure was examined during experiments using a HXP 120 mercury lamp, with FITC filter (Semrock hardcoat). Cells were voltage-clamped at a holding potential of −70 mV, after correction for a liquid junction potential of −12 mV. Mean series resistance was around 5 MΩ and was compensated up to 70% with a 10 μs lag. Presynaptic auditory nerve fibers were minimally stimulated with a monopolar electrode in a patch pipette filled with aCSF, placed at a distance of at least one cell diameter from the cell being recorded. Stimulating currents of 10–20 μA were delivered through a stimulus isolator (A360 World Precision Instruments, Sarasota, FL, USA). During recordings, bath solution (aCSF) was supplemented with: 1 mM Kynurenic acid sodium salt (abcam Biochemicals, Cambride, UK), a low-affinity AMPAR antagonist, to prevent receptor saturation/desensitization, 100 μM Cyclothiazide (CTZ; BioTrends, Wangen, Zurich), a positive allosteric AMPAR modulator, to prevent receptor desensitization, 10 μM Bicuculline methchloride, a GABA_A_ receptor antagonist and 2 μM Strychnine hydrochloride, a glycine receptor antagonist. Unless stated otherwise, chemicals were purchased from Sigma-Aldrich (St. Louis, MO, USA).

### *In Vivo* Recordings

#### Auditory Brainstem Response (ABR)

ABR is a recording of the auditory evoked potentials in response to sound stimulus, recorded from the scalp in a stimulus-locked manner. ABR waves reflect the electrical activity at different stations along the auditory pathway (Melcher et al., 1996), hence providing a measure of signal processing in the whole auditory system.

ABR recordings were performed as described earlier (Jing et al., 2013; Strenzke et al., 2016). Briefly, animals were anesthetized intraperitoneally with a combination of Ketamine (125 mg/kg) and Xylazine (2.5 mg/kg) and their core temperature was maintained at 37°C using a rectal temperature-controlled heating blanket (Hugo Sachs Elektronik; Harvard Apparatus). Additionally, their heart rate was constantly tracked to monitor the anesthesia. The electrode configuration of three subcutaneous needles was the following: the active electrode was positioned underneath the pinna, the reference electrode on the vertex and the ground electrode near the tail. For stimulus generation, presentation, and data acquisition TDT System II (Tucker-Davis Technologies) run by BioSig32 software (TDT) was used. Sound pressure levels were provided as dB SPL peak equivalent (PE) and were calibrated using a $\frac{1}{4}$ inch microphone (D 4039, Brüel and Kjaer GmbH). ABRs were obtained as an average of 2 repetitions of 2000 responses to clicks of 0.03 ms presented at 20 Hz in the free field ipsilaterally using a JBL 2402 speaker (JBL GmbH and Co.). The potential difference between vertex and mastoid subdermal needles was amplified (50,000 times), filtered (low pass: 4 kHz, high pass: 400 Hz) and sampled at a rate of 50 kHz for 20 ms. ABR threshold was determined with 10 dB precision as the lowest stimulus intensity that evoked a reproducible response waveform in both traces by visual inspection.

### Immunohistochemistry and Confocal Imaging

Animals were transcardially perfused with 2% freshly prepared ice-cold paraformaldehyde with pH adjusted to 7.4. The fixed brain was then removed and brainstem was dissected with a coronal cut few millimetres nasal to the junction between occipital cortex and cerebellum. The brain block was washed overnight in 30% sucrose solution in PBS. For sectioning, the brain block was embedded in Tissue Tek Cryomatrix (Thermo Fisher Scientific, Waltham, MA, USA) and then fixed on the stage of the cryostat (Figocut E cryotome, Reichert-Jung, Depew, NY, USA) such that the caudal aspect was facing upwards and the dorsal side was towards the blade. Advancing from caudal to nasal, 30 μm coronal sections were cut (chamber temperature: −20°C, object temperature: −22°C) and discarded until the appearance of the 7th cranial nerve. Subsequent sections containing aVCN were collected onto electrostatically charged microscope slides (SuperFrost Plus, ThermoFisher Scientific, MA, USA). For parallel processing, one slice of each genotype was collected per slide. Thereafter, the slices were washed for 10 min in PBS and incubated in Goat Serum Dilution Buffer (GSDB; 16% normal goat serum, 450 mM NaCl, 0.3% Triton X-100, 20 mM phosphate buffer, pH 7.4) for 1 h, followed by incubation in primary antibodies diluted in GSDB, for 3 h, in a wet chamber at room temperature. After washing 2 × 10 min with wash buffer (450 mM NaCl, 0.3% Triton X-100, 20 mM phosphate buffer) and 2 × 10 min with PBS, the slices were incubated with secondary antibodies diluted in GSDB for 1 h in a light-protected wet chamber at room temperature. The slices were then washed 2 × 10 min with wash buffer, 2 × 10 min with PBS and 1 × 10 min in 5 mM phosphate buffer, and finally mounted with a drop of fluorescence mounting medium based on Mowiol 4-88 (Carl Roth, Karlsruhe, Germany) and covered with a thin glass coverslip.

For inner hair cell immunohistochemistry, apical turns of freshly dissected organ of Corti were fixed in 4% formaldehyde for 10 min and processed as described for fixed brain slices.

Primary antibodies used were: rabbit anti-Piccolo (Antibody #1; 1:200), guinea pig anti-Piccolo (Antibody #2; 1:200), mouse anti-Otoferlin (1:1000; Abcam Biochemicals, Cambridge, UK), guinea pig anti-VGLUT1 (1:500), rabbit anti-VGLUT1 (1:1000), mouse anti-Gephyrin (1:500), mouse anti-Sap7f407 to Bassoon (1:500; Abcam, Cambridge, UK), guinea pig anti-Bassoon (1:500), mouse anti-Calretinin (1:300; Swant, Marly, Switzerland), guinea pig anti-VGAT (1:600), rabbit anti-Munc13-1 (1:200), rabbit anti-RIM1 (1:200), rabbit anti-RIM2 (1:200). Unless stated otherwise, primary antibodies were purchased from Synaptic Systems, Göttingen, Germany. Secondary antibodies used were: AlexaFluor488-, AlexaFluor568- and AlexaFluor647-labeled antibodies (1:200, Invitrogen).

Confocal images were acquired using a laser-scanning confocal microscope (Leica TCS SP5; Leica Microsystems) equipped with 488 nm (Ar) and 561/633 nm (He-Ne) lasers, and 63×/1.4 NA oil-immersion objective. Samples of both genotype: PicWT and PicMut were processed and imaged in parallel, using same laser power, gain and microscope settings.

### Electron Microscopy

Animals were transcardially perfused using Karlsson-Schultz buffer (Karlsson and Schultz, 1965; 4% paraformaldehyde, 2.5% glutaraldehyde, 0.5% NaCl, with pH adjusted to 7.4), and the isolated brain was fixed for an additional hour in Karlsson-Schultz buffer and subsequently 150 μm thick parasagittal slices from cochlear nuclei were obtained as described for *in vitro* physiology, except that the sections were cut at a blade feed rate of 1 mm/s and an amplitude of 1 mm, and a separate vibratome (Leica VT 1200S) was used for sectioning fixed tissue. Slices were fixed overnight on ice using 2% glutaraldehyde in 0.1 M sodium cacodylate buffer, pH 7.2 and washed three times in sodium cacodylate buffer. Post-fixation was performed for 1 h with 1% osmium tetroxide (in 0.1 M sodium cacodylate buffer), followed by a 1 h washing step in sodium cacodylate buffer and three brief washing steps in distilled water. En bloc staining using 1% uranyl acetate in distilled water for 1 h on ice was followed by a brief washing step with distilled water. Finally, slices were dehydrated at room temperature with increasing ethanol concentrations and infiltrated in Epoxy resin (Agar 100 kit, Plano, Wetzlar, Germany; 100% EtOH/Epoxy 1:1, 30 and 90 min; 100% Epoxy, overnight). Slices were further incubated in fresh 100% Epoxy resin and placed on embedding molds. After polymerization for 48 h at 70°C, 70–75 nm sections were cut and placed on formvar-coated copper slot grids (Athene, Plano, Wetzlar, Germany). Slices were post-fixed and stained with uranyl acetate replacement solution/lead citrate following standard protocols. Micrographs were taken with a JEOL electron microscope (JEM 1011, JEOL, Freising, Germany) equipped with a GatanOrius 1200A camera (Gatan, Munich, Germany) using the Gatan Digital Micrograph software package at 12,000×. Endbulb AZs of the aVCN were chosen by the appearance of the vesicles (round) and the synaptic site (asymmetric, denoted as PSD). Only excitatory synapses were taken into account. For analysis, only those AZs were used that showed a clear pre- and postsynaptic membrane.

### Data Analysis

Electrophysiology data were analyzed using Igor Pro (Wavemetrics, Lake Oswego, OR, USA), Mini Analysis (Synaptosoft Inc., Fort Lee, NJ, USA) and GraphPad Prism software (La Jolla, CA, USA). Confocal images were analyzed using ImageJ software, Imaris (Bitplane AG, Zurich, Switzerland) and Matlab (Mathworks). Endbulb terminals were tracked and counted visually using ImageJ from Calretinin-stained confocal image stacks. Figures were assembled for display using Adobe Illustrator (Adobe Systems, Munich, Germany). Statistical significance between groups was determined by either unpaired Student’s *t*-test (in case of normally distributed data with comparable variances between the groups) or Wilcoxon rank sum test (when data distribution did not satisfy the criteria). Normality of distribution was tested with Jarque-Bera test and variances were compared with F-test. Data were presented as mean ± SEM when compared using Student’s *t*-test. In case of Wilcoxon’s rank sum test, data were presented as box and whisker plots showing grand median (of the means of all recordings), lower/upper quartiles, 10–90th percentiles). *, **, *** indicate *p* < 0.05, 0.01 and 0.001 respectively.

## Results

### Perturbation of Piccolo Expression in Central but Not Peripheral Auditory Synapses

**Figure 2:**
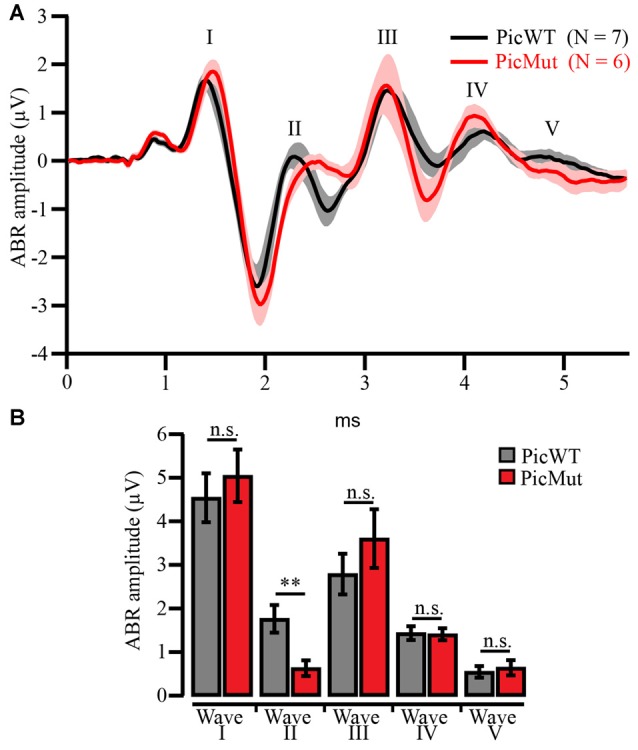
Auditory brainstem response (ABR) indicates preserved cochlear function but alteration in signal propagation at the cochlear nucleus in PicMut mice. **(A)** Grand averages (line) ± SEM (shaded area) of ABR waveform responses to 80 dB click stimuli at a stimulus rate of 20 Hz of PicWT (*N* = 7) and PicMut (*N* = 6) mice at P21–23. *N* is the number of animals. **(B)** Comparison of ABR wave amplitudes (calculated as the amplitude difference between peak of the wave and the following trough). In PicMut, wave I (compound action potential of spiral ganglion) has unaltered amplitude, but wave II thought to arise from activity in the cochlear nucleus demonstrates reduced amplitude. Interestingly, the later waves arising from downstream stations in the auditory pathway seem unaltered. Bar plots represent mean ± SEM (error bars). n.s. *p*-value ≥ 0.05, **p-value < 0.01, Student’s *t*-test.

This study employed constitutive Piccolo mutants with a targeted deletion of exon 14 of *Pclo* gene and insertion of neomycin resistance cassette in the adjacent 3′ intron, described previously (hereafter dubbed “PicMut”), which lowers the protein levels of Piccolo to approximately 5% in the brain (Mukherjee et al., 2010). A shorter, ~330 kD, C-terminal truncated *Pclo* splice variant, Piccolino (Figure 1A), is the predominant *Pclo* isoform at the ribbon synapse of IHC (Regus-Leidig et al., 2013), directly preceding the endbulb of Held. Labeling with antibody #2 (directed against a central epitope, Figure 1A), we found that IHC ribbon synapses in PicMut still express Piccolino (Figure 1C), while the full-length variant, Piccolo, was absent (identified by antibody #1 directed against a C-terminal epitope, Figures 1A,C’). Recordings of ABR (ABR; see “Materials and Methods” section) showed normal sound thresholds and unaltered amplitudes of the spiral ganglion compound action potential, reflected in wave I of the ABRs, indicating normal cochlear sound encoding (Figure 2). In contrast to IHCs, both the excitatory AZs of the endbulb of Held synapse and inhibitory AZs, in the aVCN showed a near complete loss of Piccolo staining (10% of control levels; *p*-value < 0.001, Wilcoxon rank sum test) indicating a major reduction of Piccolo expression at these central auditory synapses (Figures 1D–E). Our data also indicated a lower fluorescent intensity for Piccolo at PicWT inhibitory AZs as compared to PicWT excitatory endbulb AZs (*p*-value < 0.01, Wilcoxon rank sum test), suggesting that Piccolo may be differentially expressed in the two types of AZs. However, in this study we focus exclusively on the excitatory endbulb synapses and the exploration of Piccolo’s role at inhibitory synapses would require an independent study. The excitatory AZs at the BCs were identified by co-localization of Piccolo with vesicular glutamate transport 1 (Vglut1; maximal center of mass distance 0.4 μm in xy and 1.2 μm in z), while inhibitory AZs were distinguished by co-localization with Gephyrin (maximal center of mass distance 0.3 μm in xy and 0.9 μm in z). Immunofluorescence intensities of Vglut1 (Figure 1F; *p*-value ≥ 0.05, Wilcoxon signed rank test) and Gephyrin remained unchanged (Figure 1F’; *p*-value ≥ 0.05, paired Student’s *t*-test). We note that this did not strictly differentiate AZs of endbulbs from those of glutamatergic bouton endings, which, however, are much fewer in number (Nicol and Walmsley, 2002; Gómez-Nieto and Rubio, 2009). Therefore, and since Vglut1 is primarily associated with terminals of SGNs (Heeringa et al., 2016), our estimates were strongly dominated by endbulb AZs. Functionally, wave II of the ABRs, thought to arise from activity in the cochlear nucleus (Melcher and Kiang, 1996), demonstrated reduced amplitude suggesting a functional impairment of synaptic transmission from SGNs to the aVCN neurons (Figure 2). Interestingly, later ABR waves seemed unaltered suggesting compensation of Piccolo deficiency.

### Changes in Molecular Composition of the Active Zone Upon Piccolo Disruption

**Figure 3:**
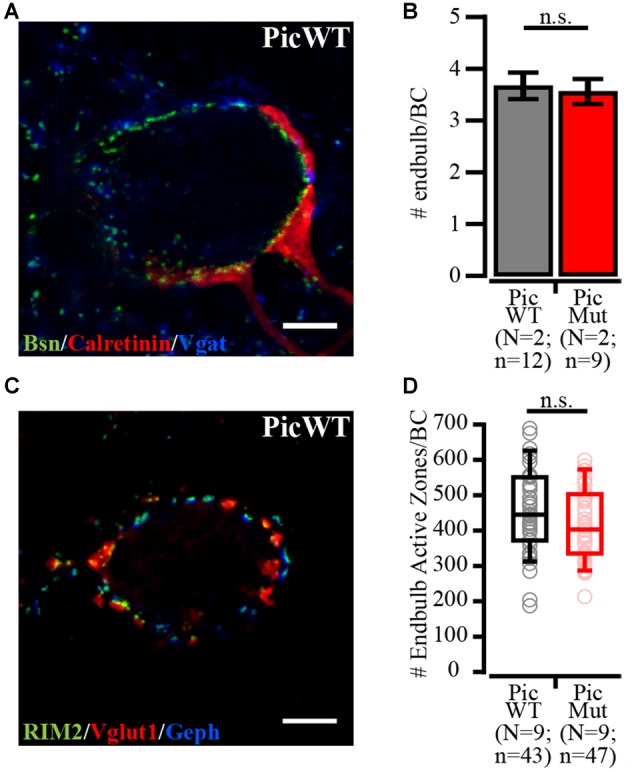
Number of endbulbs and endbulb AZs per bushy cell in aVCN. **(A)** Confocal section of a bushy cell in PicWT labeled with Bassoon (Bsn; AZ marker), Calretinin (endbulbs of Held) and Vgat (inhibitory presynaptic terminals). **(B)** Number of endbulbs converging onto a bushy cell was quantified by visually tracing and counting Calretinin-stained endbulbs. PicWT (*N* = 2; *n* = 12) and PicMut (*N* = 2; *n* = 9) receive comparable number of endbulbs (n.s. *p*-value ≥ 0.05, Student’s *t*-test). **(C)** Confocal section of a bushy cell in PicWT labeled with RIM2 (AZ marker), Vglut1 (excitatory synapses) and Gephyrin (Geph, inhibitory synapses). **(D)** Number of endbulb AZs (approximated from the # of excitatory AZs) per bushy cell quantified by subtracting the number of inhibitory AZs (AZ marker puncta juxtaposed with Gephyrin) from the total number of AZ marker puncta. Endbulb AZ number in PicWT (*N* = 9; *n* = 43) and PicMut (*N* = 9; *n* = 47) was comparable (n.s. *p*-value ≥ 0.05, Wilcoxon rank sum test). Data information: Box and whisker plot presents median, lower/upper quartiles and 10–90th percentiles. Bar plot represents mean ± SEM (error bars). *N*, number of animals; *n*, number of BCs.

We performed further semi-quantitative immunohistochemistry to analyze the effect of Piccolo disruption on the number of the endbulb of Held synapses and their AZs, as well as on the molecular composition of the AZs. We quantified the number of endbulbs converging on to BCs by visually tracing and counting Calretinin-stained endbulbs (Figure 3A; Lohmann and Friauf, 1996; Mendoza Schulz et al., 2014), not differentiating between globular and spherical BCs. BCs of both genotypes received 3-4 endbulbs on average. 3.56 ± 0.24 for PicMut and 3.67 ± 0.26 for PicWT, Figure 3B; *p*-value ≥ 0.05, Student’s *t*-test, which agrees with the number reported in the literature (Cao and Oertel, 2010; Mendoza Schulz et al., 2014) for mice after onset of hearing (p15–p21).

Next, we quantified the number of excitatory AZs per endbulb. In stacks of confocal sections of BCs (Figure 3C), we counted puncta immunofluorescent for the AZ markers (such as Bassoon, RIM1, RIM2 or Munc13-1), which gave us the total AZ count. Subtracting the number of immunofluorescent puncta juxtaposed with Gephyrin immunofluorescence (inhibitory AZ number, see above) from the total count of AZs yielded the number of excitatory AZs, which was unaltered in the PicMut synapses. 428.40 ± 17.93; (median 403) for PicMut and 455.16 ± 18.66; (median 445) for PicWT, Figure 3D, *p*-value ≥ 0.05, Wilcoxon’s rank sum test. Dividing this number by the average count of endbulbs gave us the number of AZs per endbulb which was also comparable between the genotypes (120.34 for PicMut and 124.14 for PicWT) and agreed with previous reports (Nicol and Walmsley, 2002; Mendoza Schulz et al., 2014). Hence there was no discernible change in the convergence of endbulbs to BCs or the number of AZs therein.

**Figure 4:**
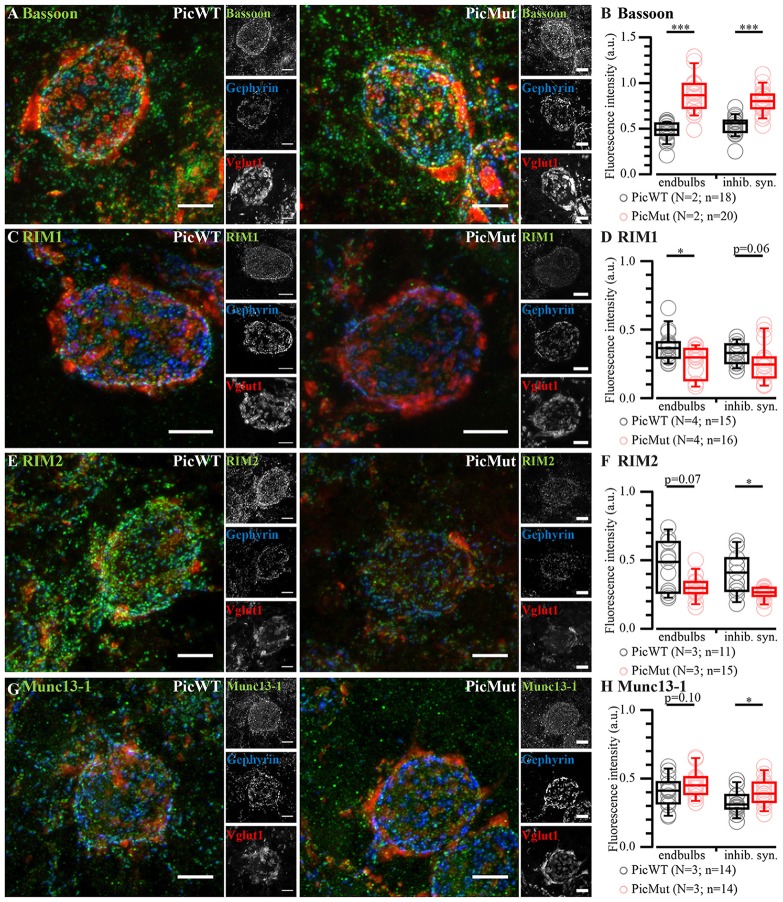
Altered molecular composition at aVCN synapses in PicMut. **(A,C,E,G)** Maximal projection of confocal image stacks of BCs in PicWT (left) and PicMut (right). Slices were immunolabeled for different CAZ proteins: Bassoon **(A)**, RIM1 **(C)**, RIM2 **(E)** and Munc13-1 **(G)** and co-stained for Vglut1 (excitatory synapses) and Gephyrin (inhibitory synapses). **(B,D,F,H)** Quantification of fluorescence intensity of CAZ proteins at endbulbs and (inhibitory synapses) of BCs: Bassoon fluorescence intensity **(B)** was significantly increased at AZs of both endbulbs and inhibitory synapses in the mutant. RIM1 **(D)** fluorescence intensity was significantly lower at the endbulb AZs in mutant but only tended to be lower at inhibitory AZs. RIM2 **(F)** fluorescence intensity tended to be reduced at all AZs, but this reached significance only at inhibitory AZs. Munc13-1 **(H)** fluorescence intensity tended to be slightly increased, which reached significance only at inhibitory AZs. Data information: *N*, number of animals; *n*, number of BCs. All scale bars −5 μm. All data presented as box and whisker plots (median, lower/upper quartiles, 10–90th percentiles). Statistical significance between groups was determined by either unpaired Student’s *t*-test (in case of normally distributed data with comparable variances between the groups) or Wilcoxon rank sum test (when data distribution did not satisfy the criteria). Normality of distribution was tested with Jarque-Bera test and variances were compared with F-test. *p-value < 0.05, ***p-value < 0.001. PicWT and PicMut samples were strictly treated in parallel and images were acquired in parallel, using same laser power, gain and microscope settings.

To study the changes in molecular composition, we quantified the immunofluorescence intensities of the CAZ proteins Bassoon, RIM1, RIM2 and Munc13-1. All CAZ proteins exhibited a spot-like fluorescence pattern around the BCs depicting AZs. Bassoon, a close homolog of Piccolo (Fenster et al., 2000), demonstrated significantly increased immunofluorescence intensity at both excitatory endbulbs and inhibitory synapses (Figures 4A,B) in PicMut mice. Immunofluorescence intensities of RIM1 and RIM2 seemed overall weaker in PicMut mice, whereby RIM1 fluorescent intensity was significantly reduced at endbulb AZs and RIM2 at AZs of inhibitory synapses (Figures 4C–F). Munc13-1 immunofluorescence was slightly higher at AZs of inhibitory synapses in PicMut mice, while the intensity was not altered at endbulb AZs (Figures 4G,H). Since the discussion on the changes observed at inhibitory AZs is beyond the scope of this study, we focused mainly on the changes observed at excitatory endbulb AZs. We conclude that Piccolo disruption leads to a reduction in the abundance of RIM1 at AZs of endbulb synapses while Bassoon is upregulated, potentially as a compensatory mechanism.

### Reduced Synaptic Vesicle Complement at Piccolo-Deficient Active Zones

**Figure 5:**
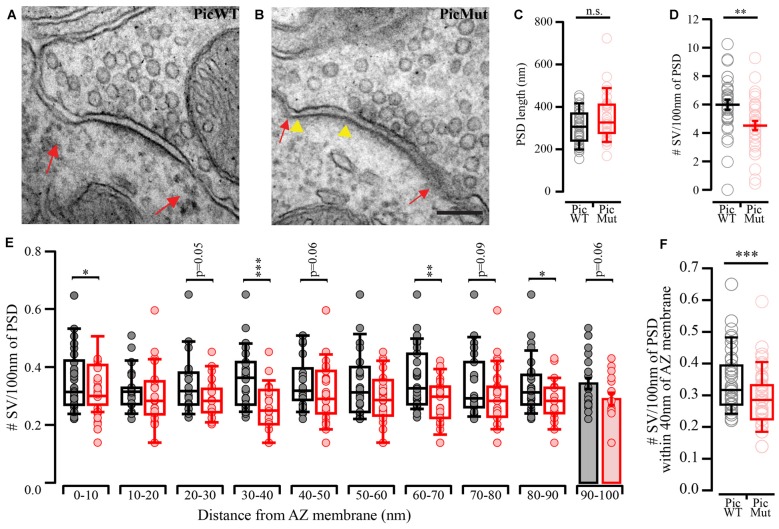
Ultrastructural analysis reveals reduced synaptic vesicle (SV) complement at PicMut AZ. **(A,B)** Representative electron micrographs of PicWT **(A)** and PicMut **(B)** endbulb synapse active zones (AZs) demonstrating reduced SVs in the mutant AZ. Red arrows demarcate the boundaries of the AZ. Yellow arrow heads mark a region of the PicMut AZ almost devoid of SVs. **(C)** Unaltered postsynaptic density (PSD) length in PicMut endbulb AZs. Box and whisker plots present grand median, lower/upper quartiles, 10–90th percentiles. n.s.—*p*-value ≥ 0.05, Wilcoxon’s rank sum test. Each data point represents the PSD length of individual endbulb AZs. **(D)** Mean SV number per 100 nm of the PSD length significantly reduced at the PicMut endbulb AZs. Data represented as mean ± SEM (**p-value < 0.01, Student’s *t*-test). Each data point represents the mean SV number per 100 nm of PSD for each endbulb AZ imaged. **(E)** Mean SV number within 0–10, 10–20, 20–30, 40–50, till 90–100 nm of the AZ perpendicular to the AZ membrane into the presynaptic cytosol normalized to PSD length. Normality of distribution was tested with Jarque-Bera test and variances were compared with F-test. Non-normally distributed data are presented as box and whisker plots, lower/upper quartiles, 10–90th percentile; *p-value < 0.05, **p-value < 0.01, ***p-value < 0.001 and were tested by Wilcoxon rank sum test. Normally distributed data were tested by Student’s *t*-test and are presented as bar plot and error bars represent SEM. **(F)** Mean SV number within 40 nm of the AZ membrane (perpendicular to the AZ membrane into the presynaptic cytosol) normalized to the length of the PSD reduced in PicMut endbulb AZs. Box and whisker plots present grand median, lower/upper quartiles, 10–90th percentiles. ***p-value < 0.001, Wilcoxon rank sum test. Each data point represents the SV number per 100 nm of PSD within 40 nm for each endbulb AZ imaged. PicWT (*N* = 3; *n* = 32) in black and PicMut (*N* = 2; *n* = 33) in red (*N*, number of animals; *n*, number of AZs).

Using electron microscopy, we investigated ultrastructural changes due to Piccolo deficiency at the AZs of endbulb synapses in ultrathin sections of aVCN (Figures 5A,B). While the length of the postsynaptic density (PSD) was unaltered in the mutant (Figure 5C, *p*-value ≥ 0.05, Wilcoxon’s rank sum test), the number of SVs per 100 nm of the PSD was significantly reduced at Piccolo-deficient endbulb AZs (Figure 5D, *p*-value < 0.01, Student’s *t*-test). For a more comprehensive analysis we compared the SV distribution within 100 nm of the presynaptic AZ membrane (perpendicular to the presynaptic membrane into the cytosol of the presynaptic terminal) in ten 10 nm bins. For each bin, there was a general trend towards fewer SVs per 100 nm of the PSD length in the mutant endbulb AZs (Figure 5E). To assess the number of membrane proximal SVs, which likely contribute to the functionally-defined RRP (Imig, 2013), we focused on the number of SVs per 100 nm of the PSD length within 40 nm of the presynaptic AZ membrane (perpendicular to the presynaptic membrane into the cytosol of the presynaptic terminal). We observed a significant reduction in the membrane proximal SVs in the mutant AZs (Figure 5F, *p*-value < 0.001 Wilcoxon rank sum test), suggesting a role of Piccolo in the organization and maintenance of the RRP at the AZ.

### Piccolo Disruption Reduces the Amplitude of Evoked EPSCs at the Endbulb of Held While Leaving the eEPSC Kinetics and Miniature EPSCs Unaltered

**Figure 6:**
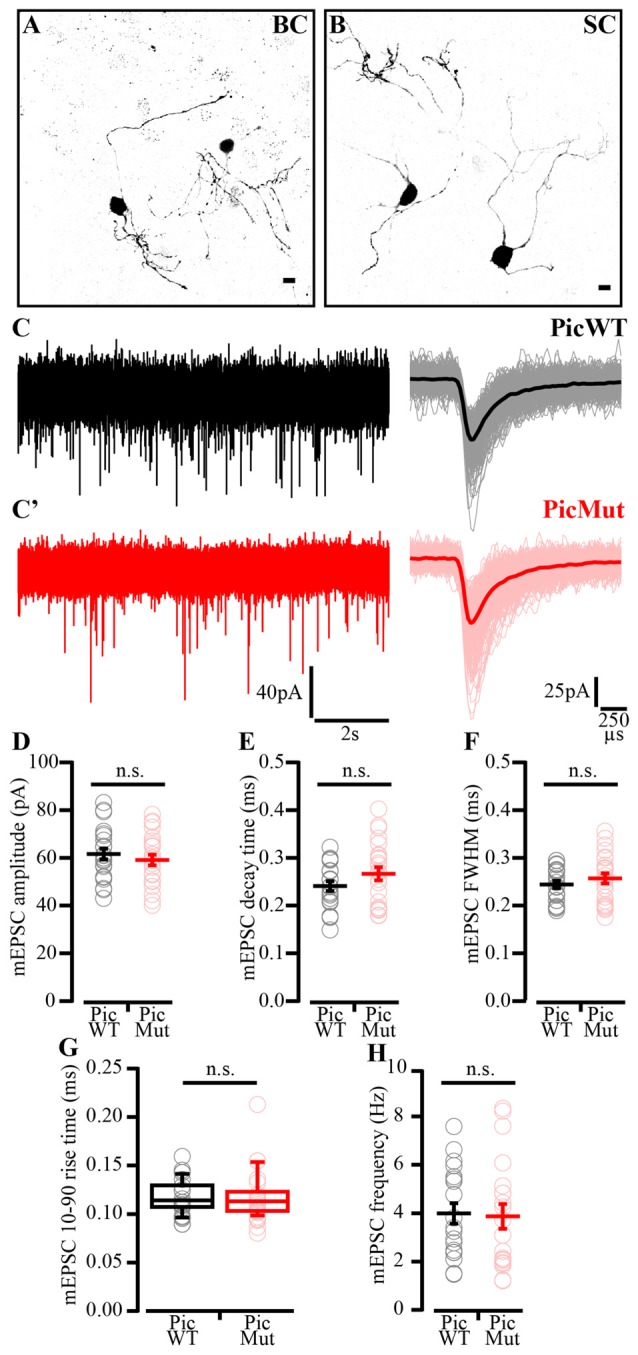
Miniature EPSC amplitude and kinetics preserved in PicMut synapses. **(A,B)** Images of bushy cells (BC, **A**) and stellate cells (SC, **B**) filled with fluorescent dye Alexa 488 and fixed after the recording, illustrating typical BC morphology, spherical with one primary dendrite ending in a dense bush-like dendritic tree and typical SC morphology, asymmetrical with multiple far-ranging dendrites branching off in different directions **(B)**. All four cells were recorded and imaged at different times and the images assembled together for presentation. **(C)** Representative traces of mEPSC: Continuous recording (left) and average (dark bold line) of all mEPSC events of the representative cell (light thin lines; right) for PicWT **(C)** and PicMut **(C’)**. **(D–H)** Analysis of mEPSC: mEPSC amplitude **(D)**, decay time **(E)**, full-width at half-maximum (FWHM; **F**) rise time **(G)** and frequency **(H)** remained unaltered. Each data point represents the mean estimate of a given BC. Normally distributed data presented as mean (grand average of the means of all BCs) ± SEM **(D–F,H**; n.s.—*p*-value ≥ 0.05, Student’s *t*-test). Non-normally distributed data presented as box and whisker plots (grand median (of the means of all BCs), lower/upper quartiles, 10–90th percentiles; **(G)** n.s.—*p*-value ≥ 0.05, Wilcoxon rank sum test). PicWT *N* = 16; *n* = 22, PicMut *N* = 16; *n* = 24 (*N*, number of animals; *n*, number of BCs).

To determine the functional consequences of Piccolo disruption at the endbulb of Held synapse, we studied synaptic transmission in acute sagittal slices of the brainstem of PicMut and PicWT mice by recording EPSCs from BCs at postnatal days 15–21. BCs were distinguished from stellate cells (SC; another major cell type in the aVCN) by the faster kinetics of their postsynaptic currents (Isaacson and Walmsley, 1995) and their characteristic short-term plasticity (Chanda and Xu-Friedman, 2010). In addition to such functional identification, each recorded cell was filled with fluorescent dye Alexa 488 via the patch pipette for morphological distinction. BCs are spherical in appearance with one primary dendrite terminating in a dense bush-like dendritic tree (Wu and Oertel, 1984), distinct from SC, which are asymmetrical in shape and have multiple dendrites branching off in various directions giving them a star-like appearance (Figures 6A,B).

We first studied miniature EPSCs (mEPSC), in the presence of 1 mM Kynurenic acid (Elmslie and Yoshikami, 1985) and 100 μM CTZ (Yamada and Tang, 1993), to check if quantal size or kinetics of single vesicle release were altered at Piccolo-deficient endbulbs. As previously reported (Oleskevich and Walmsley, 2002), application of TTX does not alter the spontaneous EPSCs (sEPSCs) at the mouse BCs. This has been corroborated by various other studies (Lu et al., 2007; Mendoza Schulz et al., 2014). Therefore, mEPSCs were recorded as spontaneous events in whole-cell recordings of BC in Pic WT (Figure 6C) and PicMut (Figure 6C’) that were voltage-clamped at −70 mV (Figure 6). We did not observe differences in the mEPSC amplitude (Figure 6D), kinetics (Figures 6E–G) and frequency (Figure 6H; *p*-value ≥0.05 for all three quantities, Figures 6D–F,H Student’s *t*-test, Figure 6G Wilcoxon rank sum test). To eliminate the possibility of CTZ obscuring the changes in AMPA receptor composition, we compared the decay kinetics of mEPSC events in the absence of CTZ. The mEPSC decay times for PicWT (157.10 ± 2.86 μs; median 158 μs) and PicMut (161.72 ± 3.27 μs; median 155 μs) were comparable (*p*-value ≥ 0.05, Wilcoxon’s rank sum test).

**Figure 7:**
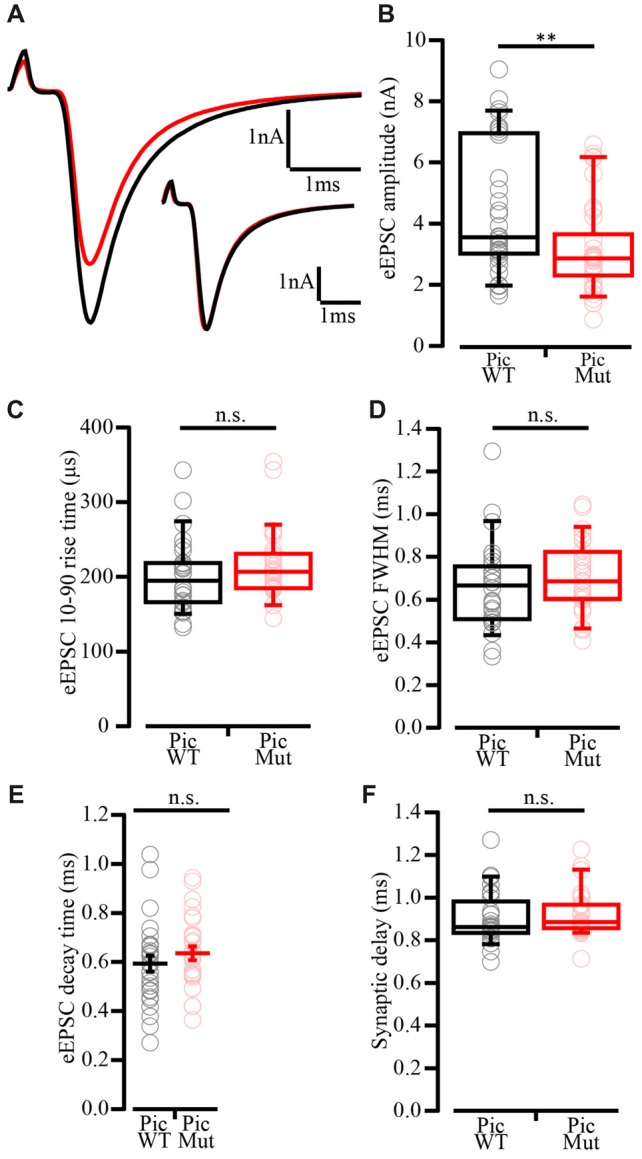
Reduced evoked EPSC amplitude in Piccolo-deficient endbulb of Held synapses. **(A)** Average traces of evoked EPSC (eEPSC) in PicWT (black) and PicMut (red) showing reduced eEPSC amplitude in the mutant. Inset: Average PicMut eEPSC trace scaled to the peak of the average wildtype trace demonstrating unaltered eEPSC kinetics in the mutant. Positive peak at onset of trace reflects the stimulation artifact. **(B)** Reduced eEPSC amplitude in PicMut (*N* = 19; *n* = 34) as compared to PicWT (*N* = 21; *n* = 33). Each data point represents the mean estimate of a given BC, box and whisker plots present grand median (of the means of all BCs), lower/upper quartiles, 10–90th percentiles). **p-value < 0.01, Wilcoxon rank sum test. **(C–F)** eEPSC kinetics: rise time **(C)**, full-width at half-maximum (FWHM; **D**) and decay time **(E)**, and synaptic delay **(F)** were not significantly altered between the two genotypes. Non-normally distributed data presented as box and whisker plots (grand median (of the means of all BCs), lower/upper quartiles, 10–90th percentiles; **(C,D,F)** n.s. —*p*-value ≥ 0.05, Wilcoxon rank sum test). Normally distributed data presented as mean (grand average of the means of all BCs) ± SEM (**E**; n.s.—*p*-value ≥ 0.05, Student’s *t*-test). PicWT *N* = 19; *n* = 28, PicMut *N* = 19; *n* = 28 (*N*, number of animals; *n*, number of BCs).

Next, we investigated evoked synaptic transmission in the presence of 1 mM Kynurenic acid and 100 μM CTZ to avoid saturation and desensitization of AMPA receptors (Chanda and Xu-Friedman, 2010), respectively. Evoked EPSCs (eEPSC) were elicited by minimal electrical stimulation of the auditory nerve fibers by a monopolar electrode placed in the proximity of the recorded BC, whereby each stimulus is aimed to elicit one action potential in one endbulb (Yang and Xu-Friedman, 2008). Analysis of eEPSC evoked by single stimulations (Figure 7) revealed reduced eEPSC amplitude in BCs of PicMut mice (3.15 ± 0.25 nA; median 2.83 nA) as compared to PicWT (4.43 ± 0.37 nA; median 3.52 nA), Figures 7A,B, *p*-value < 0.01, Wilcoxon rank sum test. The kinetics of rise and decay (Figures 7C–E), and the synaptic delay (Figure 7F) remained unaltered; *p*-value ≥ 0.05, Figures 7C,D,F Wilcoxon’s rank sum test and Figure 7E Student’s *t*-test. Synaptic delay was calculated as the time between the start of stimulus (voltage output of the amplifier as dictated by the experiment protocol) and the time when the respective EPSC response reaches 10% of its peak amplitude.

### Reduced RRP Size and a Slower Recovery from Short-Term Depression in Piccolo Mutants

**Figure 8:**
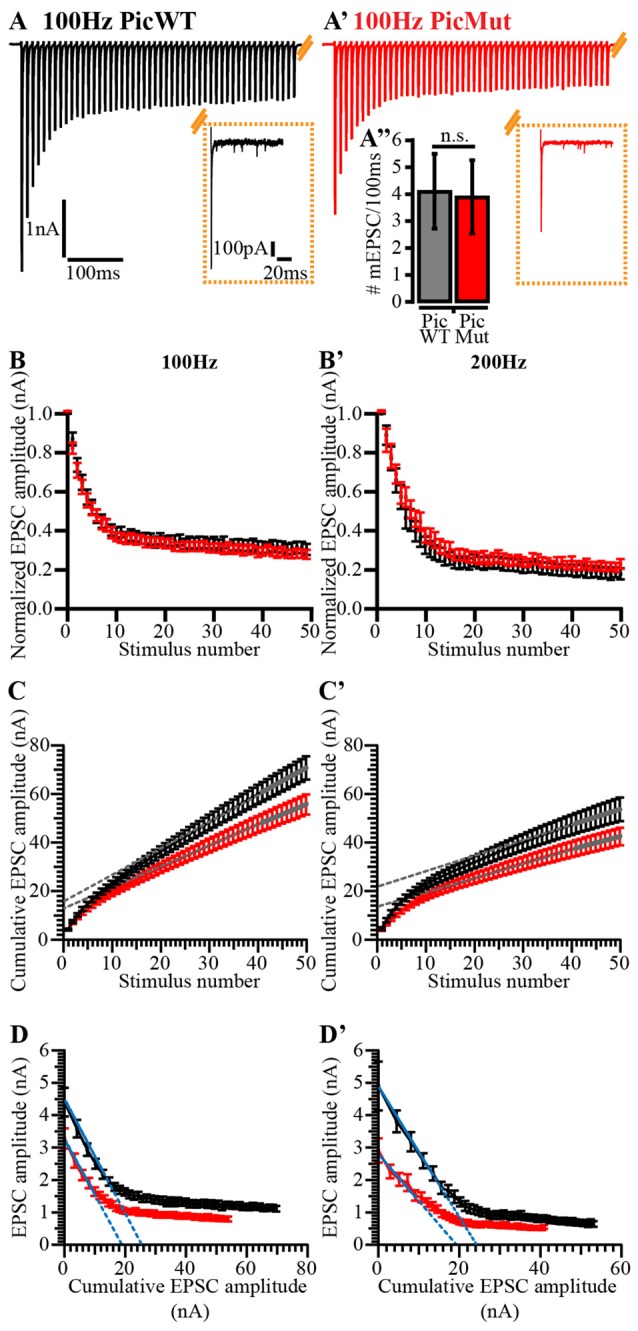
Analyzing vesicle pool dynamics during high-frequency stimulation at Piccolo-deficient endbulb of Held synapses. **(A)** Average traces of EPSCs evoked in response to 100 Hz train stimulation, recorded from PicWT **(A)** and PicMut **(A’)** endbulb synapses, illustrating characteristic fast kinetics and short-term depression of bushy cell EPSCs, which remain preserved in the mutant. Inset shows the last EPSC of the train and spontaneous activity (mEPSC events) for 100 ms after the cessation of train stimulus, demonstrating comparable asynchronous activity in PicWT (black, left) and PicMut (red, right) (**A”**; PicWT *N* = 7; *n* = 9, PicMut *N* = 6; *n* = 10) *p*-value ≥ 0.05, Student’s *t*-test. **(B,B’)** Normalized EPSC amplitude plotted against stimulus number demonstrates comparable short-term depression in PicWT (black) and PicMut (red) in response to high-frequency train stimulation at 100 Hz **(B)** and 200 Hz **(B’)**. **(C,C’)** To estimate the readily releasable pool size (RRP), replenishment rate and release probability (P_r_) using the Schneggenburger-Meyer-Neher (SMN) method, EPSCs from trains were plotted cumulatively against stimulus number and the linear fit (solid gray line) to the last ten steady-state amplitudes was back-extrapolated (dotted gray line) to the y-axis for 100 Hz **(C)** and 200 Hz **(C’)**. For quantitative analysis refer to Table 1. **(D,D’)** To estimate the RRP and P_r_ using the Elmqvist and Quastel (EQ) method, absolute EPSC amplitudes from trains were plotted against cumulative amplitudes of the all EPSCs preceding the corresponding EPSC and, the linear fit (solid blue line) to the first 3–5 data points was forward-extrapolated (dotted blue line) to the x-axis for 100 Hz **(D)** and 200 Hz **(D’)**. For quantitative analysis refer to Table 1. For 100 Hz: PicWT *N* = 19; *n* = 28, PicMut *N* = 19; *n* = 28. For 200 Hz: PicWT *N* = 13; *n* = 21, PicMut *N* = 13; *n* = 19. *N*, number of animals; *n*, number of BCs.

Quantal size (mEPSC amplitude) being unaltered, we next investigated the RRP size, release probability and pool dynamics to investigate the cause of the reduced eEPSC amplitude. We studied responses to high frequency train stimulation: 50 consecutive stimuli delivered at 100 and 200 Hz (in the presence of 1 mM Kynurenic acid and 100 μM CTZ). At these frequencies, both PicMut and PicWT mice exhibited comparable short-term depression (Figures 8A,A’). Both genotypes demonstrated similar kinetics of short-term depression during train stimulation (Tau (τ), Table 1; Figures 8B,B’). The paired pulse ratio (PPR) was not significantly altered at inter-stimulus intervals of 5 ms and 10 ms (Table 1). The extent of depression assessed as the amplitude in the steady-state reached after 30 train pulses was comparable (EPSC_30–50_/EPSC_1_, Table 1; Figures 8B,B’).

**Table 1:** Comparison of RRP size, vesicle replenishment, release probability and short-term depression at the endbulb of Held synapse in PicWT and PicMut mice.

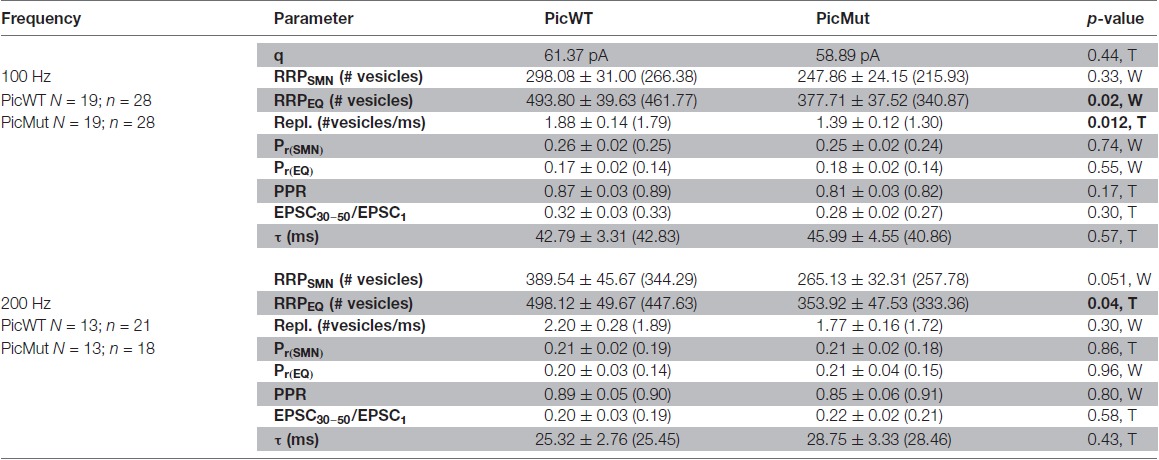

*q: quantal size, taken from mEPSC amplitude; RRP: readily releasable pool; Repl.: replenishment rate of vesicles; P_r_: release probability; EPSC_30–50_/EPSC_1_: average amplitude of EPSC30–50 normalized to the amplitude of EPSC1; τ: time constant of a single exponential fit to the decay of evoked EPSC amplitudes during train stimulation. Data presented as mean ± SEM, and medians in parentheses. Statistical significance between groups was determined by either unpaired Student’s t-test (in case of normally distributed data with comparable variances between the groups, T) or Wilcoxon rank sum test (when data distribution did not satisfy the criteria, W). Normality of distribution was tested with Jarque-Bera test and variances were compared with F-test. *p*-value < 0.05 signifying statistical significance in bold*.

The size of the RRP, the release probability (P_r_) and the SV replenishment rate were estimated by applying two variants of the cumulative analysis to the EPSC trains: Schneggenburger-Meyer-Neher (SMN) method (Schneggenburger et al., 1999) and Elmqvist and Quastel (EQ) method (Elmqvist and Quastel, 1965; both recently reviewed in Neher, 2015). In the SMN method (Figures 8C,C’), EPSC amplitudes from trains are plotted cumulatively against the stimulus number. A line fit to the steady-state points (last 10 of the 50 points) is back-extrapolated to the y-axis, and the y-intercept divided by the mEPSC amplitude estimates the RRP size, while P_r_ is estimated by the ratio of vesicle content of EPSC_1_ to that of the RRP. The slope of the linear fit itself, approximates the rate of vesicle replenishment during the train. The SMN analysis (Figures 8C,C’, Table 1) only revealed a non-significant trend towards a reduced RRP size in the mutant. Another finding was the reduced rate of vesicle replenishment in the mutant that reached significance at 100 Hz stimulation (*p*-value = 0.012, Student’s *t*-test). The estimate of P_r_ was not significantly changed upon Piccolo disruption at any stimulation frequency. Asynchronous release following the train stimulation was unchanged: the rate of mEPSCs was not different in first 100 ms after the end of 100 Hz trains between BCs of both genotypes (Figure 8A” *inset*).

In the EQ method (Figures 8D,D’ used previously at central auditory synapse in Taschenberger et al., 2002), absolute EPSC amplitude in response to a given stimulus, n (EPSC_n_), in the train is plotted against the cumulative EPSC amplitudes of the stimuli prior to stimulus n (Cumulative amplitudes of EPSC_0_ to EPSC_n−1_). A line fit to the first 3–5 points is forward extrapolated to the x-axis and the x-intercept divided by the mEPSC amplitude estimates the RRP size, while P_r_ is determined as the slope of the linear fit. In contrast to the SMN method, the EQ method more robustly reported the reduced RRP size in Piccolo mutant endbulb synapses (Figures 8D,D’, Table 1), demonstrated by a significant reduction in RRP at both 100 and 200 Hz stimulation frequencies. As with the SMN method, the estimates of P_r_ were comparable between the two genotypes.

**Figure 9:**
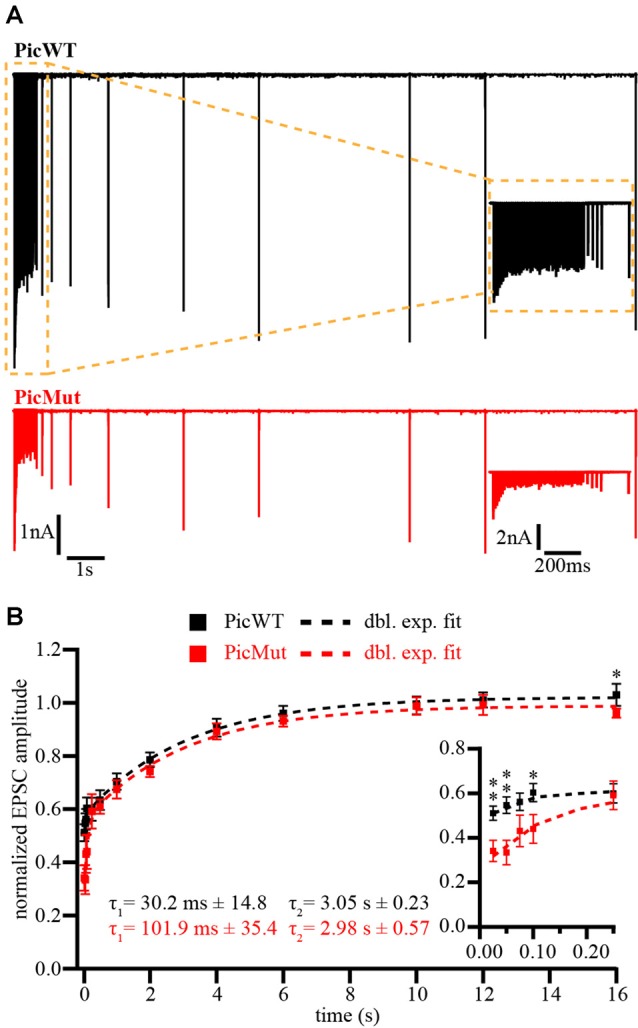
Recovery from short-term depression is slowed at Piccolo-deficient endbulb of Held synapses. **(A)** Representative traces of PicWT (upper panel, black) and PicMut (lower panel, red) endbulb synapses to illustrate the recovery protocol. Following a conditioning 100 Hz train of 50 stimuli, recovery from short-term depression was assessed by single test pulses presented after (in ms) 25, 50, 75, 100, 250, 500 (further in s) 1, 2, 4, 6, 10, 12 and 16. Inset shows the responses to the first 5 stimuli in detail. **(B)** Recovery plotted as mean (± SEM) EPSC amplitude in response to test pulses normalized to the first EPSC amplitude of the conditioning train. Dashed lines are double exponential fits. The time constants (τ) are provided on the graph, amplitude ratios of the two recovery components (fast/slow) were 0.15 and 0.55 for PicWT and PicMut respectively. Inset shows the first five responses in detail. **p-value < 0.01, *p-value < 0.05. Statistical significance between groups was determined by either unpaired Student’s *t*-test (in case of normally distributed data with comparable variances between the groups) or Wilcoxon rank sum test (when data distribution did not satisfy the criteria). Normality of distribution was tested with Jarque-Bera test and variances were compared with F-test. PicWT *N* = 15; *n* = 10–18, PicMut *N* = 25; *n* = 12–24. *N*, number of animals; *n*, number of BCs.

To further verify our finding of slower vesicle replenishment in PicMut endbulbs at 100 Hz suggested by SMN analysis, we studied the recovery from short-term depression, by measuring eEPSC amplitudes elicited by single stimuli presented at varying time intervals after a conditioning 100 Hz train of 50 pulses (Figure 9A). Recovery is displayed as the eEPSC amplitudes normalized to the amplitude of the first eEPSC of the conditioning train (Figure 9B). The time course of recovery was fitted with a double exponential function:

f(x)=y0−A1e−Δtτ1−A2e−Δtτ2

A double exponential time course of recovery at physiological temperatures has been previously reported for calyceal synapses: endbulb of Held (Yang and Xu-Friedman, 2008) and calyx of Held (Kushmerick et al., 2006), with time constants comparable to our wildtype estimates. The amplitude and tau (τ) values for the fits were as follows: for PicMut A_1_ = 0.24 ± 0.04, τ_1_ = 101.9 ms ± 35.4; A_2_ = 0.43 ± 0.03, τ_2_ = 2.98 s ± 0.57 and PicWT A_1_ = 0.07 ± 0.01, τ_1_ = 30.2 ms ± 14.8; A_2_ = 0.44 ± 0.01, τ_2_ = 3.05 s ± 0.23. PicMut endbulbs showed a significantly slower recovery during the initial phase (Figure 9B) with longer time constant for the fast component of the double exponential fits. The kinetics of the slow components were comparable.

### In the Absence of Piccolo, Partial Loss of Bassoon Affects Evoked Transmission, Short-Term Depression and Recovery from Depression at the Endbulb of Held, but Has No Effect on Spontaneous Release

**Figure 10:**
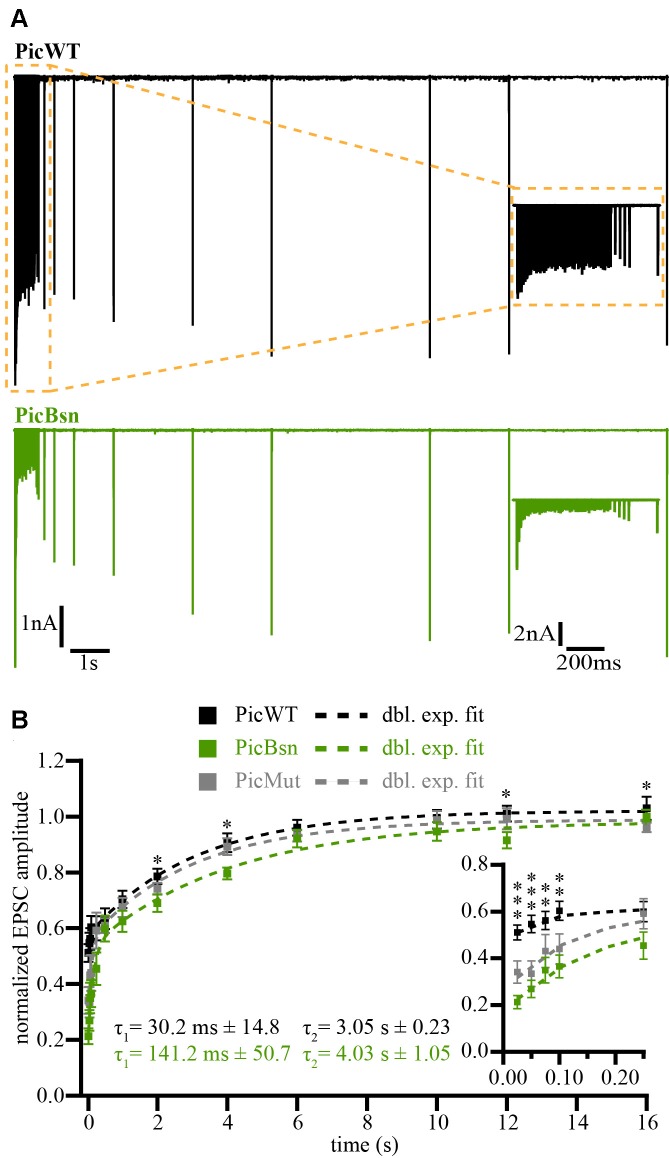
Impairment in recovery from short-term depression further aggravated at PicBsn endbulb of Held synapses. **(A)** Representative traces of PicWT (upper panel, black) and PicBsn (lower panel, green) endbulb synapses to illustrate the recovery protocol. Following a conditioning 100 Hz train of 50 stimuli, recovery from short-term depression was assessed by single test pulses presented after (in ms) 25, 50, 75, 100, 250, 500 (further in s) 1, 2, 4, 6, 10, 12 and 16. Inset shows the responses to the first 5 stimuli in detail. **(B)** Recovery plotted as mean (± SEM) EPSC amplitude in response to test pulses normalized to the first EPSC amplitude of the conditioning train. Dashed lines are double exponential fits. The time constants (τ) are provided on the graph, amplitude ratios of the two recovery components (fast/slow) were 0.15 and 0.67 for PicWT and PicBsn respectively. PicMut recovery trace shown in gray for comparison. Inset shows the first five responses in detail. Comparing PicWT and PicBsn recoveries, ***p-value < 0.001, **p-value < 0.01, *p-value < 0.05. Statistical significance between groups was determined by either unpaired Student’s *t*-test (in case of normally distributed data with comparable variances between the groups) or Wilcoxon rank sum test (when data distribution did not satisfy the criteria). Normality of distribution was tested with Jarque-Bera test and variances were compared with F-test. PicWT *N* = 15; *n* = 10–18, PicBsn *N* = 5; *n* = 10. *N*, number of animals; *n*, number of BCs.

In addition to the mutation of Piccolo, we also studied the effect of disrupting (deleting exons 4 and 5, *Bsn*^ΔEx4/5^, Altrock et al., 2003) one allele of Bassoon on vesicle replenishment rate. The mutant is henceforth referred to as PicBsn. Using the recovery paradigm described above, we estimated the kinetics of SV recovery after short-term depression by means of double exponential fits (Figure 10). The amplitude and tau (τ) values of the fit at PicBsn synapses were as follows: A_1_ = 0.30 ± 0.04, τ_1_ = 141.2 ms ± 50.7; A_2_ = 0.45 ± 0.04, τ_2_ = 4.03 s ± 1.05. In previous studies (Mendoza Schulz et al., 2014), heterozygosity for Bsn^ΔEx4/5^ did not have any phenotype on synaptic transmission. However, in the absence of Piccolo, even partial loss of Bassoon seems to exacerbate the slowing of vesicle replenishment (Figure 10B).

The aggravation of slowed recovery from vesicle depletion upon Bassoon disruption in addition to Piccolo mutation, provided the impetus to investigate synaptic transmission in PicBsn mice. For a comprehensive analysis all three modes of release (Kaeser and Regehr, 2014): spontaneous, asynchronous and evoked, were studied, just like for PicMut mice. All recordings were made in the presence of 1 mM Kynurenic acid and 100 μM CTZ to eliminate postsynaptic factors influencing the inference.

**Figure 11:**
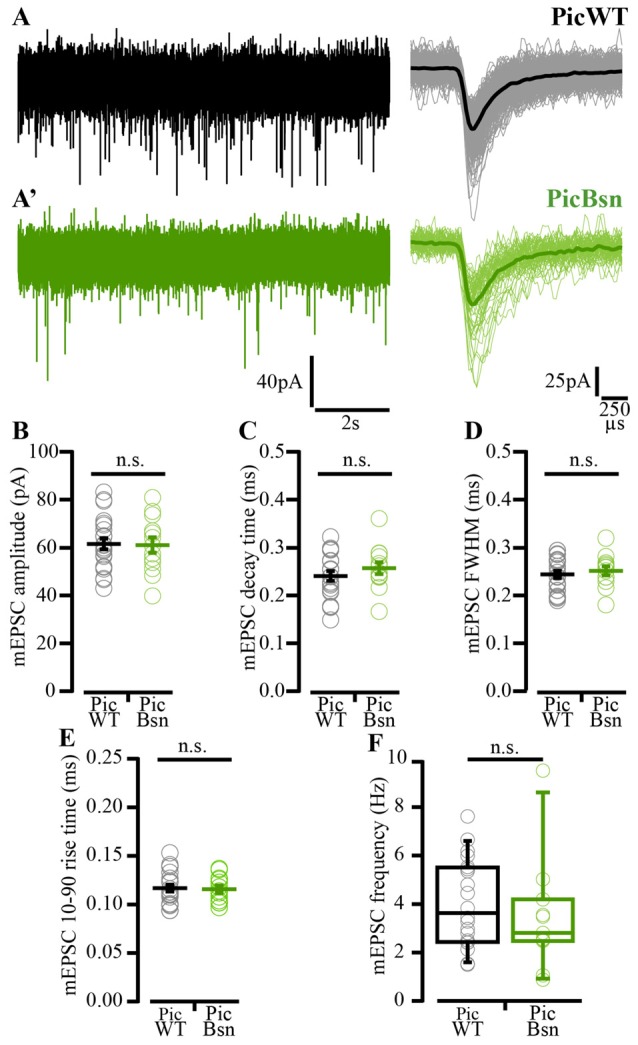
Miniature EPSC amplitude and kinetics preserved in PicBsn endbulb synapses. **(A)** Representative traces of mEPSC: Continuous recording (left) and average (dark bold line) of all mEPSC events of the representative cell (light thin lines) (right) for PicWT **(A)** and PicBsn **(A’)**. **(B)** Analysis of mEPSC: mEPSC amplitude **(B)**, decay time **(C)**, full-width at half-maximum (FWHM; **D**), rise time **(E)** and frequency **(F)** remain unaltered. Each data point represents the mean estimate of a given BC. Normally distributed data presented as mean (grand average of the means of all BCs) ± SEM (**B–E**; n.s. *p*-value ≥0.05, Student’s *t*-test). Non-normally distributed data presented as box and whisker plots (grand median (of the means of all BCs), lower/upper quartiles, 10–90th percentiles; **(F)** n.s.—*p*-value ≥ 0.05, Wilcoxon rank sum test). PicWT *N* = 16; *n* = 22, PicBsn *N* = 5; *n* = 13. *N*, number of animals; *n*, number of BCs.

mEPSC recorded as spontaneous events from postsynaptic BCs were comparable between PicWT (Figure 11A) and PicBsn (Figure 11A’). The analysis did not reveal any differences in the mEPSC amplitude, kinetics or frequency of events (*p*-value ≥ 0.05 for all three quantities, Figures 11B–E Student’s *t*-test, Figure 11F Wilcoxon rank sum test).

**Figure 12:**
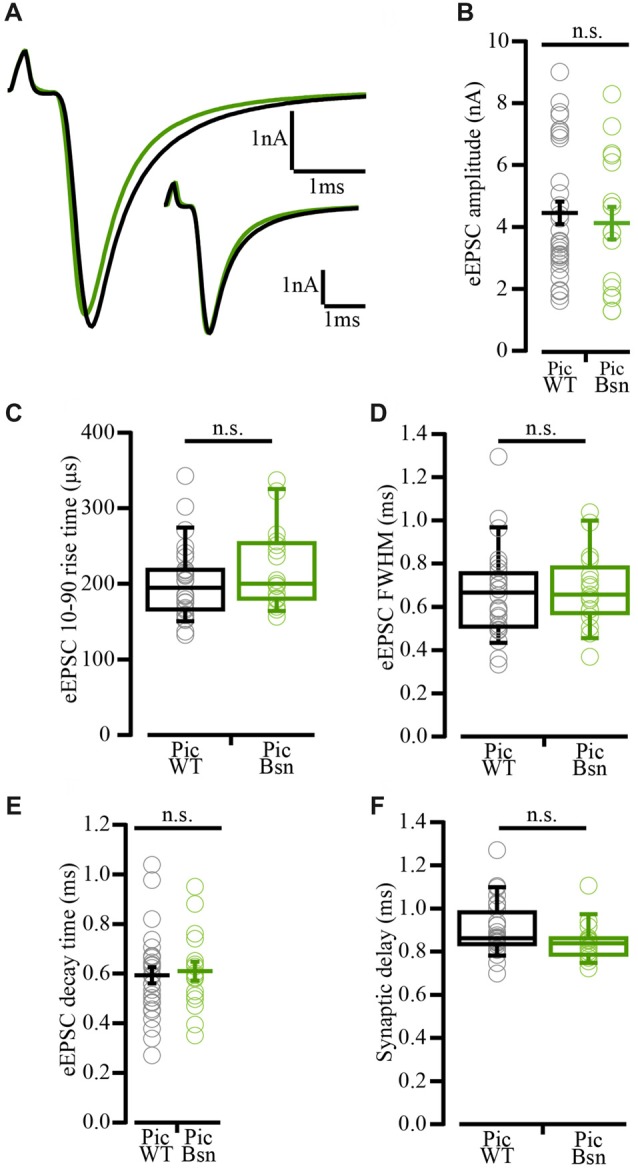
Unaltered evoked EPSC amplitude in PicBsn mutants as “pseudo-rescue” of PicMut phenotype. **(A)** Average traces of evoked EPSC (eEPSC) in PicWT (black) and PicBsn (green) showing unaltered eEPSC amplitude in the PicBsn mice. Inset: Average PicBsn eEPSC trace scaled to the peak of the average wildtype trace demonstrating unaltered eEPSC kinetics in the mutant. Positive peak at onset of trace reflects the stimulation artifact. **(B)** Unaltered eEPSC amplitude in PicBsn (*N* = 6; *n* = 17) as compared to PicWT (*N* = 21; *n* = 33). Data presented as mean (grand average of the means of all BCs) ± SEM (n.s.—*p*-value ≥ 0.05, Student’s *t*-test). **(C–F)** eEPSC kinetics: rise time **(C)**, full-width at half-maximum (FWHM; **D**) and decay time **(E)**, and synaptic delay **(F)** were not significantly altered in PicBsn as compared to PicWT. Non-normally distributed data presented as box and whisker plots (grand median (of the means of all BCs), lower/upper quartiles, 10–90th percentiles; **(C,D,F)** n.s. *p*-value ≥ 0.05, Wilcoxon rank sum test). Normally distributed data presented as mean (grand average of the means of all BCs) ± SEM (**E**; n.s. *p*-value ≥ 0.05, Student’s *t*-test). PicWT *N* = 19; *n* = 28, PicBsn *N* = 6; *n* = 17. *N*, number of animals; *n*, number of BCs.

Interestingly, the reduced eEPSC amplitude phenotype observed in Piccolo-deficient synapses showed a “pseudo-rescue” upon additional deletion of a single Bassoon allele. eEPSC amplitudes were largely unaltered in PicBsn endbulbs (Figures 12A,B; 4.10 ± 0.25 nA for PicBsn vs. 4.43 ± 0.37 nA for PicWT, *p*-value ≥ 0.05, Student’s *t*-test). The kinetics of rise and decay (Figures 12C–E), and the synaptic delay (Figure 12F) remained unaltered; *p*-value ≥ 0.05, Figures 12C,D,F Wilcoxon’s rank sum test and Figure 12E Student’s *t*-test. Again, in contrast to PicMut, endbulbs of PicBsn mice demonstrated a lower PPR in response to pairs of stimuli presented at inter-stimulus intervals of 5 and 10 ms (Table 2).

**Table 2:** Comparison of RRP size, vesicle replenishment, release probability and short-term depression at the endbulb of Held synapse in PicWT and PicBsn mice.

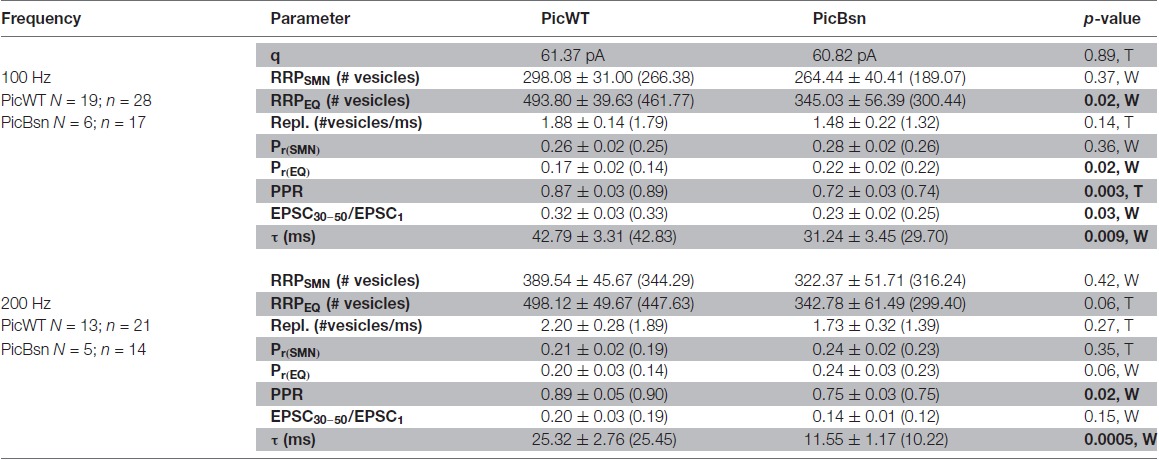

*q: quantal size, taken from mEPSC amplitude; RRP: readily releasable pool; Repl.: replenishment rate of vesicles; P_r_: release probability; EPSC_30–50_/EPSC_1_: average amplitude of EPSC 30–50 normalized to the amplitude of EPSC1; τ: time constant of a single exponential fit to the decay of evoked EPSC amplitudes during train stimulation. Data presented as mean ± SEM, and medians in parentheses. Statistical significance between groups was determined by either unpaired Student’s t-test (in case of normally distributed data with comparable variances between the groups, T) or Wilcoxon rank sum test (when data distribution did not satisfy the criteria, W). Normality of distribution was tested with Jarque-Bera test and variances were compared with F-test. *p*-value < 0.05 signifying statistical significance in bold*.

**Figure 13:**
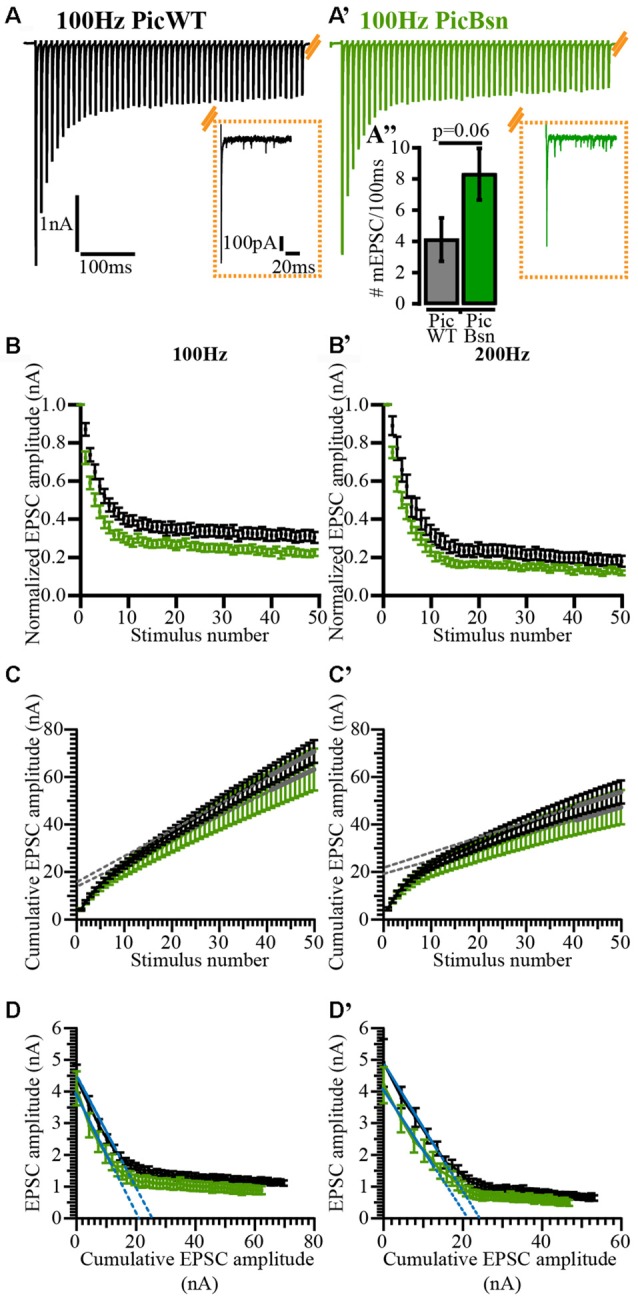
Analyzing vesicle pool dynamics during high-frequency stimulation at PicBsn endbulb of Held synapses. **(A)** Average traces of EPSCs evoked in response to 100 Hz train stimulation, recorded from PicWT **(A)** and PicBsn **(A’)** endbulb synapses, illustrating characteristic fast kinetics and short-term depression of bushy cell EPSCs, which remain preserved in the mutant. Inset shows the last EPSC of the train and spontaneous activity (mEPSC events) for 100 ms after the cessation of train stimulus, demonstrating a trend towards increased asynchronous activity in PicBsn (green, right) as compared to PicWT (black, left) (**A”**; PicWT *N* = 7; *n* = 9, PicBsn *N* = 4; *n* = 13) *p*-value = 0.06, Student’s *t*-test. **(B,B’)** Normalized EPSC amplitude plotted against stimulus number demonstrates greater short-term depression in PicBsn (green) as compared to PicWT (black) in response to high-frequency train stimulation at 100 Hz **(B)** and 200 Hz **(B’)**. **(C,C’)** To estimate the readily releasable pool size (RRP), replenishment rate and release probability (P_r_) using the SMN method, EPSCs from trains were plotted cumulatively against stimulus number and the linear fit (solid gray line) to the last ten steady-state amplitudes was back-extrapolated (dotted gray line) to the y-axis for 100 Hz **(C)** and 200 Hz **(C’)**. For quantitative analysis refer to Table 2. **(D,D’)** To estimate the RRP and P_r_ using the EQ method, absolute EPSC amplitudes from trains were plotted against cumulative amplitudes of the all EPSCs preceding the corresponding EPSC, and the linear fit (solid blue line) to the first 3–5 data points was forward-extrapolated (dotted blue line) to the x-axis for 100 Hz **(D)** and 200 Hz **(D’)**. For quantitative analysis refer to Table 2. For 100 Hz: PicWT *N* = 19; *n* = 28, PicBsn *N* = 6; *n* = 17. For 200 Hz: PicWT *N* = 13; *n* = 21, PicBsn *N* = 5; *n* = 14. *N*, number of animals; *n*, number of BCs.

A lower PPR is indicative of a higher release probability which was not seen in PicMut mice. To further analyze the effects of Bassoon disruption in addition to the absence of Piccolo, responses to high frequency train stimulations were studied (Figures 13A,A’) to assess the RRP size, release probability and pool dynamics. The evoked response was quantified using cumulative analysis as described above (Figures 13C–D’; Table 2). Undetected in the SMN analysis, the EQ analysis revealed a smaller RRP and an increased P_r_ in the PicBsn endbulbs as compared to PicWT (Table 2). Short-term depression was also stronger at endbulbs of PicBsn mice than in PicWT as determined by a smaller τ (time constant of decay of the response to train stimulation) and a reduced steady-state amplitude (EPSC_30–50_/EPSC_1_, significant only at 100 Hz; Figures 13B,B’, Table 2). This contrasts with the phenotype observed with Piccolo mutation alone, where no discernible change in short-term depression was noted. Endbulb of Held synapses in the PicBsn mice tended to have more asynchronous activity as compared to PicWT synapses, but this did not reach statistical significance (Figure 13A” *inset*).

## Discussion

We studied the role of Piccolo in high frequency transmission at the endbulb of Held synapses of the auditory pathway. Piccolo disruption elicited changes in the molecular composition of the AZ, some of which might partially compensate for Piccolo deficiency (Bassoon) while the reduction of RIM1 might contribute to the observed deficit in vesicle pool size (Han et al., 2011). Through electrophysiology, we provide evidence for a role of Piccolo in maintaining the RRP size and efficiency of recovery from short-term depression. Additionally, we aimed to segregate the function of Piccolo from that of its close homolog, Bassoon. We propose that Piccolo and Bassoon promote efficient SV-replenishment to the RRP in an additive manner and differ regarding their impact on release probability.

### Changes in Molecular Composition of Endbulb AZs Upon Piccolo Disruption

Deletion of exon 14 of *Pclo* along with insertion of a neomycin resistance cassette drastically reduced Piccolo expression as observed by semi-quantitative analysis by immunofluorescence. The reduction of Piccolo immunofluorescence on average amounted approximately to 90%, which is close to what was found by Western blotting of whole brain homogenate (95%; Mukherjee et al., 2010). Therefore, manipulation of Piccolo employed at the endbulb synapse was incomplete and, hence, the analysis likely underestimated the role of the protein. This calls for a refined genetic deletion that targets all splice variants. However, this drawback of the currently used *Pclo* manipulation also offered an advantage: the C-terminus, affected by the mutation, is absent from its shorter variant, Piccolino (Regus-Leidig et al., 2013, 2014), the predominant Piccolo isoform at the ribbon synapses of the IHCs. The data illustrate that the Piccolino expression remains intact at the IHCs of PicMut. ABR recordings corroborated the notion of intact IHC synapses and indicated overall normal cochlear function. This provided a unique opportunity to selectively study the effects of Piccolo disruption at the endbulb without any bias from the preceding synapse that was confounding the previous analysis of Bassoon function at the endbulb synapse (Mendoza Schulz et al., 2014).

The gross morphology of the endbulb synapse was not affected by Piccolo disruption: the number of the auditory nerve fibers converging onto BCs and the number of AZs per endbulb remained unaltered. Since Piccolo interacts directly or indirectly with most other CAZ proteins (Wang et al., 2009), we also studied how the molecular composition of AZ is affected upon its disruption. Semi-quantitative expression analysis was employed by integrating immunofluorescence of excitatory and inhibitory AZs onto BCs. Since excitatory AZs were required to co-localize with Vglut1 that is thought to distinguish endbulb terminals from Vglut2-positive bouton endings (Heeringa et al., 2016), it is reasonable to consider the analysis of excitatory AZs to mostly report properties of endbulb AZs. Out of the four CAZ proteins (Bassoon, RIM1, RIM2 and Munc13-1) investigated, expression levels of Bassoon and RIM1 were significantly altered at endbulb AZs. It is interesting to note that the two proteins that were altered share regions of homology with Piccolo (Wang et al., 1999). RIM and Piccolo both exhibit Zn finger domains, a PDZ domain and two C-terminal C_2_ domains and Bassoon shares ten highly conserved PBH domains with Piccolo (tom Dieck et al., 1998; Wang et al., 1999; Fenster et al., 2000). RIM1 immunofluorescence intensity was reduced while that of Bassoon was increased. It can be speculated that the reduction in RIM1 could be a consequence of it not being integrated properly at the synapse in the absence of Piccolo, as Piccolo is thought to play an integral role in AZ assembly and scaffolding (Gundelfinger et al., 2016). Bassoon, on the other hand, thought to have partially overlapping functions with Piccolo (Mukherjee et al., 2010; Waites et al., 2013), could be upregulated in PicMut as a compensatory mechanism. This is in line with a previous report (Mendoza Schulz et al., 2014), where Piccolo expression was upregulated in Bassoon deficient synapses, likely to compensate for the loss of some of Bassoon’s function.

### Piccolo Disruption Mildly Affects Synaptic Transmission at the Endbulb of Held

So far, a unifying picture of the role of Piccolo in synaptic transmission has been missing. While one study implicated Piccolo as a negative regulator of SV exocytosis (Leal-Ortiz et al., 2008), others stated Piccolo plays no major role in SV release (Mukherjee et al., 2010) or, together with Bassoon, contributes to maintenance of the structural integrity of the AZ (Waites et al., 2013). In the present study of the endbulb of Held synapse, an amplitude reduction of eEPSCs evoked by single action potentials was observed. BCs are responsible for high fidelity transmission of timing information (Oertel, 1999). Hence, even this mild reduction (~20%) in eEPSC amplitudes at the level of individual synapses potentially seems to impair the synchronized activity in the cochlear nucleus as demonstrated by the reduction of ABR wave II amplitude.

Kinetics of release, on the other hand, were not changed either for eEPSCs or for mEPSCs. The unaltered mEPSCs amplitude (quantal size) and kinetics rule out post-synaptic changes in abundance or properties of AMPA receptors. This is supported by the unchanged spatial extent of the PSD in PicMut endbulb AZs, reported by the electron micrograph analysis of the synaptic ultrastructure. In contrast, global disruption of Bassoon increased the quantal size and significantly enlarged the PSD length (Mendoza Schulz et al., 2014). This was suggested to reflect homeostatic upscaling of excitatory synaptic contacts in response to reduced SGN input to BCs due to impaired synaptic sound encoding in the cochlea. Excluding postsynaptic alterations and changes in vesicular glutamate content based on the unaltered mEPSCs, leaves two possibilities, a reduction in either the release probability (P_r_) or the RRP size to explain the reduced eEPSC amplitude. The EQ analysis of eEPSC trains (Elmqvist and Quastel, 1965) indicated a reduced RRP size as the primary reason for the reduced eEPSC amplitude. While, the SMN (Schneggenburger et al., 1999) method failed to disclose a significant reduction in RRP size, our notion was corroborated by a reduced synaptic vesicle pool at the AZ observed on an ultrastructural level using electron microscopy. P_r_, on the other hand, seemed largely unaltered as estimated by both the analysis methods.

After cessation of the stimulus following depression, the recovery protocol revealed an impairment in SV recovery, where the fast phase of recovery was slowed by a factor of three. Piccolo’s contribution to vesicle replenishment could be attributed to its interaction with Actin regulatory protein like Daam1, required for F-actin assembly (Wagh et al., 2015), that may regulate conversion of reluctant SVs to the fast releasing RRP (Lee et al., 2012). Moreover, the reduction in RIM1 might contribute to the observed reduction in RRP, as RIMs play a crucial role in making vesicles fusion competent (Wang et al., 1997; Gracheva et al., 2008; Deng et al., 2011; Han et al., 2011; Fernández-Busnadiego et al., 2013; Kintscher et al., 2013; Jung et al., 2015) and hence in determining the RRP size (Han et al., 2011, 2015). With its extended reach into the cytosol, tens of nanometers beyond the dense projections of the CAZ (Limbach et al., 2011), Piccolo might escort SVs from further away in the presynaptic cytosol and deliver them to RIM to make them release ready. The reduction in RIM1 expression reported in this study could be in line with this synaptic interplay. Indeed, our electron microscopy analysis demonstrating a reduced pool of SVs at the Piccolo-deficient AZs lends support to this hypothesis.

Interestingly, a trend towards further slowing of the fast phase of recovery from short-term depression was observed upon disrupting one allele of Bassoon, in addition to the mutation of Piccolo. Nevertheless, finding reduced vesicle replenishment upon disruption of either Bassoon (Hallermann et al., 2010; Mendoza Schulz et al., 2014) or Piccolo suggests that neither protein alone is sufficient to maintain wildtype performance of SV replenishment, even despite a likely compensatory, up-regulation of the respective other gene.

This implies partially additive function, probably even at the same AZs as the majority of them contain both proteins (Dondzillo et al., 2010; Mendoza Schulz et al., 2014), although Piccolo and Bassoon may promote replenishment through different molecular pathways. As discussed above, Piccolo might employ an interplay of F-actin assembly, interaction with other CAZ proteins like RIM and its Ca^2+^ sensing domain. Bassoon on the other hand, might tether SVs via interactions with the SV associated protein Mover (Ahmed et al., 2013; Körber et al., 2015) and could also assist in positioning of Ca^2+^ channels (Davydova et al., 2014) and SVs close to each other (Hallermann et al., 2010).

Future work will be required to dissect the precise molecular mechanisms by which Piccolo acts in vesicle replenishment. This should also target roles of Piccolo in SV endocytosis and low-affinity Ca^2+^ sensing. Piccolo binds Actin associated proteins like Abp1 (Fenster et al., 2003), and GIT1 (Kim et al., 2003), both of which have been linked to SV endocytosis. As stated earlier, Piccolo plays a role in dynamic assembly of F-actin, which is needed for retrieval of SVs both by bulk (Nguyen et al., 2012) and ultrafast (Watanabe et al., 2013) endocytosis. The finding that only the fast component of recovery was affected would imply a rapid mechanism, such as ultrafast endocytosis or release-site clearance (Haucke et al., 2011), to be involved. The C_2_A domain of Piccolo acts as a low- affinity Ca^2+^ sensor (Gerber et al., 2001; Garcia et al., 2004), which is ideal to sense Ca^2+^ build-up during high-frequency activity at the synapse. At the endbulb of Held, Ca^2+^ drives the fast component of recovery (Wang and Manis, 2008; Yang and Xu-Friedman, 2008) and Piccolo is an interesting candidate as the sensor driving this Ca^2+^ dependent rapid recovery.

### Segregation of the Roles of Piccolo and Bassoon at the Active Zone

To elucidate the synergistic function of Bassoon and Piccolo, physiology of Piccolo mutant mice with additional heterozygosity for a partial deletion of the Bassoon gene (PicBsn mice, Bsn: deletion of exons 4 and 5; Altrock et al., 2003), was further probed. The phenotype observed was intermediate between the changes in neurotransmission seen in Piccolo and Bassoon-deficient endbulbs (Mendoza Schulz et al., 2014). Bassoon heterozygosity did not exert any synaptic deficits by itself (Mendoza Schulz et al., 2014). However, at the endbulb of Held, in the absence of Piccolo, Bassoon heterozygotes demonstrate a reduced PPR indicative of an increased release probability, faster and greater extent of depression to train stimulation, and a tendency for higher asynchronous release upon cessation of high frequency stimulation.

Interestingly, the eESPC amplitude, which was reduced in Piccolo-deficient endbulbs, seemed comparable to the PicWT values in PicBsn mice demonstrating a “pseudo-rescue” of PicMut phenotype. In Bassoon KO, the eEPSC amplitude was comparable to that of the wildtype likely because of homeostatic upscaling of excitatory synaptic contacts in response to reduced SGN input to BCs due to impaired synaptic sound encoding in the cochlea (Mendoza Schulz et al., 2014). However, this is not valid for PicBsn mice, as the cochlea does not suffer from Piccolo deficiency and one allele of Bassoon seems sufficient to sustain neurotransmission at the cochlear ribbon synapses (Frank et al., 2010). This is reflected in the unaltered mEPSC amplitude, as opposed to an increase observed at endbulbs of Bassoon KO (Mendoza Schulz et al., 2014).

EQ analysis, but not SMN analysis, estimated a reduction in RRP. This discrepancy in the estimations of the two methods arises from the assumptions on which they are based. The SMN analysis estimates the pool size by the decrement in the vesicle pool during stimulation and is thus influenced by the extent of depression during the train (Neher, 2015). Hence, an increased depression (lower steady-state amplitude) at PicBsn endbulbs could overestimate RRP size giving values, comparable to that of the PicWT. EQ analysis on the other hand, estimates the RRP by forward-extrapolation, as it would have been before the onset of stimulation and is hence independent of the stimulation frequency used and the synaptic depression resulting therein (Neher, 2015). Considering the reduced RRP at the PicBsn synapses, we speculate that the “pseudo-rescue” of eEPSC amplitude is due to an increase in release probability as indicated by a lower PPR and higher P_r_ by the EQ cumulative analysis.

Taken together, partial lack of Bassoon in Piccolo-deficient synapses seems to add changes in the release probability to the phenotype observed in PicMut mice. It is interesting to speculate that Piccolo and Bassoon maintain synaptic vesicle clustering at the synapse (Mukherjee et al., 2010), but while they both have a collaborative role in vesicle replenishment, Bassoon might have a role in regulation of Ca^2+^-influx, SV release and/or their coupling as well. This hypothesis opens questions and a need for further investigations to probe the changes in interactions and compositions of the CAZ proteins and the active zone ultrastructure in the absence of two of its largest proteins.

## Author Contributions

This study was conceived by TM and TB. The experimental work was performed by TB (acute slice electrophysiology, immunohistochemistry, contribution to electron microscopy) and CW (electron microscopy). TB and TM prepared the manuscript with contributions of CW.

## Conflict of Interest Statement

The authors declare that the research was conducted in the absence of any commercial or financial relationships that could be construed as a potential conflict of interest.
